# Variations in the Relative Abundance of Gut Bacteria Correlate with Lipid Profiles in Healthy Adults

**DOI:** 10.3390/microorganisms11112656

**Published:** 2023-10-28

**Authors:** Ineta Kalnina, Dita Gudra, Ivars Silamikelis, Kristine Viksne, Ance Roga, Edmunds Skinderskis, Davids Fridmanis, Janis Klovins

**Affiliations:** Latvian Biomedical Research and Study Centre 1, LV-1067 Riga, Latvia

**Keywords:** cholesterol, hyperlipidaemia, gut microbiome, metagenome, *Akkermansia muciniphila*, *Massilistercora timonensis*

## Abstract

The gut microbiome is a versatile system regulating numerous aspects of host metabolism. Among other traits, variations in the composition of gut microbial communities are related to blood lipid patterns and hyperlipidaemia, yet inconsistent association patterns exist. This study aims to assess the relationships between the composition of the gut microbiome and variations in lipid profiles among healthy adults. This study used data and samples from 23 adult participants of a previously conducted dietary intervention study. Circulating lipid measurements and whole-metagenome sequences of the gut microbiome were derived from 180 blood and faecal samples collected from eight visits distributed across an 11-week study. Lipid-related variables explained approximately 4.5% of the variation in gut microbiome compositions, with higher effects observed for total cholesterol and high-density lipoproteins. Species from the genera *Odoribacter*, *Anaerostipes*, and *Parabacteroides* correlated with increased serum lipid levels, whereas probiotic species like *Akkermansia muciniphila* were more abundant among participants with healthier blood lipid profiles. An inverse correlation with serum cholesterol was also observed for *Massilistercora timonensis*, a player in regulating lipid turnover. The observed correlation patterns add to the growing evidence supporting the role of the gut microbiome as an essential regulator of host lipid metabolism.

## 1. Introduction

Since the introduction of “omics” approaches more than a decade ago, the human gut microbiome has been recognized as a critical player in the regulation of metabolic homeostasis in the host [1]. The gut microbiome contributes to numerous aspects of host metabolism by converting dietary nutrients and producing active metabolites that are involved in signalling cascades that maintain host metabolic processes [2]. Although the definition of a healthy gut microbiome probably will never be introduced due to the high diversity of the system and the complexity of its interactions, specific perturbations in its composition are linked with phenotypes like metabolic syndrome, obesity, diabetes, and related metabolic diseases, including cardiovascular diseases [3,4,5].

Apart from other metabolic traits, the gut microbiome has been implicated in the development of hyperlipidaemia. Some of the early small-scale association studies have shown a correlation between decreased gut microbiome diversity and circulating lipid levels, particularly with triglyceride (TG) and high-density lipoprotein (HDL) levels, and highlighted a positive relationship between HDL and an abundance of the *Clostridium* genus among adults [6,7]. The study by Fu and colleagues [8] described novel associations between *Christensenellaceae*, *Pasteurellaceae*, *Eggerthella*, *Butyricimonas*, and circulating TG and HDL levels. Furthermore, they demonstrated that the gut microbial communities can explain, on average, 4.5% of the variation in blood lipids independently from such significant confounders as body mass index (BMI), age, gender, and genetic risk factors [8]. Since then, multiple gut microbiome taxonomic and functional features have been associated with blood lipid fractions, including circulating HDL, low-density lipoprotein (LDL) cholesterol, and TG levels, through cross-sectional studies in cohorts representing the general population [3,8,9,10,11,12,13]. Patients with hypercholesterolaemia can be recognized from normolipidaemic controls on the basis of their gut microbiome profile [5,14,15]. Examples of bacterial taxa in the human gut microbiome that have shown an association with blood lipid levels across multiple studies include representatives of the families *Bifidobacteriaceae*, *Christensenellaceae*, *Lachnospiraceae*, *Veillonellaceae*, and *Ruminococcaceae*, as well as the phylum *Actinobacteria*, particularly members of the genus *Lactobacillus* [10,11,13,15,16]. The study conducted in a Japanese population has pointed out that the gut microbiomes in patients with hypercholesterolaemia share similar characteristics with that of type 2 diabetes cases. They observed an increased abundance of the genera *Bifidobacterium* and *Collinsella* and a decreased proportion of *Bacteroides* among patients with either of these metabolic diseases [17].

Further evidence supporting the connection between the composition of gut microbial communities and host lipid metabolism comes from studies addressing the metabolic impact of the consumption of prebiotic and probiotic supplements. The intake of probiotics predominantly comprising *Lactobacillus* and *Bifidobacterium* species significantly reduced total cholesterol (total Ch) and LDL plasma levels across distinct cohorts [18,19,20,21]. Although some studies show a beneficial impact on HDL and TG levels [18,22,23], the regulatory effects of probiotics on circulating HDL and TG are less consistent across different cohorts [19,20,21]. Several associations between microbial and lipid traits were specific only to the probiotic intervention group, highlighting the importance of inter-specific interactions within the complex system of the gut microbiome [23]. Like probiotics, prebiotics are widely used to modulate the gut microbiome [18,24]. Apart from affecting lipid turnover directly by regulating the functions of the gastrointestinal tract and decreasing the bioavailability of lipids from the diet [25,26], soluble fibre reduces total Ch and LDL levels by facilitating the growth of bacteria that are described as beneficial for metabolic health [25,27]. Bacteria enriched by fibre consumption include short-chain fatty acid (SCFA) producers such as *Faecalibacterium*, *Roseburia*, *Bifidobacterium*, and *Akkermansia* [27,28].

Causal relationships between the gut microbiome and host lipid metabolism have been confirmed in animal studies. Thus, microbial communities transferred from hyperlipidaemic human donors to mice with a depleted gut microbiome replicated a phenotype characterized by high plasma cholesterol levels in recipient animals [29]. In humans, a recent Mendelian randomization analysis conducted by Guo and colleagues [30] indicated a causal link between *Actinobacteria*, *Terrisporobacter*, and serum LDL, as well as between bacteria representing the genus *Oscillospira* and circulating levels of TG.

Two primary mechanisms through which the gut microbiome can modulate the host’s cholesterol metabolism are the regulation of bile acid turnover and production of SCFAs [31,32]. Microbiome-derived SCFAs regulate multiple aspects of host metabolism. SCFAs interact with key energy sensors such as AMP-activated protein kinase (AMPK), regulate angiopoietin-like 4 (ANGPTL4) signalling protein, and bind specific G-protein coupled receptors (GPCRs), including GPR41 and GPR43. These interactions result in the modulation of signalling cascades governing appetite and energy intake via gut-derived hormones like GLP-1, to maintain glucose homeostasis and control lipid turnover [31,33]. Apart from serving as a direct energy source in the gut as well as at the systemic level, SCFAs are used as substrates in lipogenesis and gluconeogenesis [32,33]. The mechanisms responsible for the SCFA-mediated control of cholesterol metabolism include the inhibition of cholesterol transport from the intestine, increased cholesterol uptake by the liver, and the suppression of the expression of key hepatic cholesterol synthesis regulators [33,34]. Butyrate, acetate, and propionate are three major SCFAs produced by gut bacteria from dietary fibre [31,35]. Butyrate is predominantly utilized as an energy source by enterocytes. Propionate is thought to be responsible for the downregulation of cholesterol synthesis, particularly the conversion of acetate, one of the major substrates for cholesterol production [31,32,35].

Investigations of the relationships between levels of SCFAs in hosts and metabolic outcomes have thus far produced mixed results [35,36]. For example, some studies in animal models have shown that the administration of SCFAs reduced cholesterol in plasma [34,37,38]. Similarly, in human cohorts, the beneficial effects of dietary fibre on lipid profiles have been attributed to increased SCFA production [27]. However, other authors have found a positive correlation between SCFAs, obesity measures, and circulating lipids [5,36,38,39,40]. Among other findings, positive relationships were found in healthy women during pregnancy between all three dominant SCFAs, acetate, propionate, and butyrate, and major plasma lipid fractions, including HDL, LDL, and TG [39]. Since SCFAs like propionate and acetate play different roles in lipid metabolism, variations in the overall effect on lipid turnover could be determined by differences in the ratios between specific SCFAs [35]. It is also possible that the observed correlations vary along with metabolic backgrounds, changes in physiological requirements, gut microbiome compositions, and other factors [5,36,39,40].

Most cholesterol is converted into bile acids. Bile acids undergo different modifications by gut bacteria, like deconjugation, which promotes their retention in the gut lumen and excretion. The bile acid pool is then replenished by de novo synthesis, depleting cholesterol levels [31,32]. Secondary bile acids also activate Takeda GPR 5 (TGR5) and farnesoid X receptor (FXR). While TGR5 regulates GLP-1 production and glucose turnover, the activation of FXR inhibits cholesterol synthesis in the liver and stimulates cholesterol efflux back into the gut lumen and its subsequent disposal [29,31].

Other mechanisms involved in gut microbiome-regulated host cholesterol turnover include the conversion of cholesterol to coprostanol, which is an unabsorbable cholesterol metabolite, and the direct absorption of dietary cholesterol, as suggested for probiotic bacteria like *Lactobacillus* species [41,42].

Although intensive research has resulted in a description of numerous individual-specific and environmental factors shaping the gut microbiome, only 15% of the variance in the taxonomic and functional composition of the gut microbiome can be attributed to factors taken into account thus far [43]. An essential step towards developing microbiome-based approaches for precision medicine would be to assess patterns replicated across numerous studies and disentangle the complexity of microbiome-related processes. Despite numerous studies connecting the gut microbiome and the regulation of blood lipid levels, there is still high heterogeneity among the observed association patterns [11,12,13,14,30,44]. Therefore, our aim was to assess the relationships between the composition of the gut microbiome and variations in lipid profiles among healthy adults in a short-term follow-up study.

## 2. Materials and Methods

**Participants and study design.** To assess the correlations between serum lipid parameters and properties of the gut microbiome, we used data and samples donated by 23 participants within the framework of a previously conducted dietary intervention study. The dietary intervention study was implemented in 2018 from May to August to evaluate the possible impact of smoked meat products on the gut microbiome.

The study was designed as a randomized crossover trial with three parallel intervention groups, each assigned a different sequence of three study products: salmon (delivered fresh), smoked salmon, and lean smoked pork provided by a certified food supplier. The study lasted 11 weeks. The first two weeks served as an adaptation period. The adaptation period was followed by three week-long intervention periods separated by two washout periods lasting two weeks each. The last intervention period was followed by a two-week post-intervention washout period.

The study involved healthy free-living volunteers who were randomly divided into three groups, each starting with a different product (salmon, smoked salmon, or pork), and then crossing over to the next product so that the three intervention periods covered the three types of intervention diet concurrently. The study participants were given seven portions containing 150 g of the respective product at the beginning of each intervention period and instructed to eat a portion per day with their habitual diet for seven days. The products were stored in household freezers. The participants prepared fresh salmon at will at home. Besides avoiding cooking over an open fire, the cooking method was not specified. The participants were also asked to maintain their usual diet and lifestyle habits. The background diet was not controlled. A few restraints included eating fish during the intervention week and smoked, smoke-flavoured, or grilled products during the study period.

An invitation to participate in the dietary intervention study was distributed by approaching personal contacts and through social networks like Facebook. Recruitment was carried out in two rounds, separated by a three-week delay during May and at the beginning of June 2018 in collaboration with the Genome Database of the Latvian population (LGDB), which provided resources for data and sample management [45]. Participants from both study recruitment rounds followed the same protocol. The participants were required to be 18 to 64 years old and have no constraints on the consumption of animal products. The exclusion criteria were acute gastrointestinal, liver, renal, or oncological diseases, metabolic diseases such as diabetes mellitus or autoimmune diseases, pregnancy or breastfeeding, use of microbiome-altering medication (antibiotics or PPI) within the past two months, or diarrhoea within the past week. In total, 32 people responded to the invitation, and 26 fulfilled the inclusion/exclusion criteria. Two participants withdrew during the adaptation period due to health problems, and one participant left due to personal schedule changes that were incompatible with the requirements of the study. This left 23 participants, from which 21 participants completed all eight visits. Two women left the study after the fifth and seventh visits. The reasons for withdrawal were acute upper respiratory infection and enterovirus infection, respectively. The data obtained from the completed visits were retained for statistical analysis.

**Sample processing.** In total, 180 paired whole blood and faecal samples were collected. The samples were obtained during eight visits, distributed over an 11-week study period, according to the following schedule: the first visit before the two-week run-in period, followed by six visits scheduled before and after each of the three one-week intervention periods, separated by two-week washout periods, and the eighth final visit after the last two-week washout period. General phenotypic data, including age, body height, and weight, were collected using questionnaires developed by the LGDB and given to participants at the initial study visit [45]. Data on habitual diets were collected through food frequency questionnaires filled out during recruitment. The collected data were merged into 13 food categories: red meat (beef and pork), poultry, fish (including other sea products), eggs, dairy products, vegetables, potatoes, fruits, cereals (including bread, pasta, rice, and buckwheat), legumes, sugary foods (candies, chocolate, and cakes), sweetened pastries (including cookies), and beverages (including water consumption). The frequency of product use was transformed into ordinal data as follows: not consumed (1), consumed once or twice per week (2), three to four times per week (3), five to six times per week (4), and more than seven times per week (5). Five categories were used to characterize the volume of consumed beverages: less than three glasses (1), three to five glasses (2), six to eight glasses (3), more than seven glasses (4), and more than two litres of liquids consumed per day (5).

To obtain lipid profiles, whole blood samples were collected in EDTA vacutainer tubes without an anticoagulant in the morning of each visit after an overnight fast. The certified biochemical laboratory performed measurements of serum lipid levels in millimoles per litre (mmol/L) using standard colourimetric assays [45]. Non-HDL cholesterol (Non-HDL Ch) levels were calculated by subtracting the HDL from the total Ch.

Faecal samples were collected by the participants at home with a provided sample collection kit following written instructions. The collection kit contained standard polypropylene faecal collection tubes without a preservation solution (ThermoFisher Scientific, Waltham, MA, USA). The faecal samples were collected as closely as possible to the scheduled blood sample collection visit. The participants were asked to freeze and store the samples in household freezers until transportation on cold elements to the laboratory for long-term storage at −80 °C. Microbial DNA isolation was performed with a FastDNA Spin Kit for Soil (MP Biomedicals, Santa Ana, CA, USA) using ≈ 150 mg of the sample. DNA samples were quantified with a Qubit 2.0 and Qubit dsDNA HS assay kit (ThermoFisher Scientific, USA), and the average DNA yield was 172.2 ± 83.7 ng/μL. Metagenome libraries were prepared using an MGIEasy Universal DNA Library Prep Set with 250 ng of DNA as the sample input (MGI Tech Co., Ltd., Shenzhen, China). The quality of the DNA libraries was assessed with a 2100 Bioanalyzer instrument using an Agilent High Sensitivity DNA Kit (Agilent, Santa Clara, CA, USA). The composition of the gut microbiome was determined with the paired-end whole-metagenome sequencing approach on a DNBSEQ-G400 instrument following the PE100 protocol with a DNBSEQ-G400RS High-throughput Sequencing Set (FCL PE100) (MGI Tech Co., Ltd., China). Background contamination was tested throughout the process using a blank control containing ultrapure water. The total raw read count for the blank control was 3820, including 593 on-target reads. The quality of the library preparation and sequencing steps were checked using the ZymoBIOMICS Microbial Community DNA Standard (Zymo Research, Tustin, CA, USA).

**Data processing.** On average, 51,058,661 ± 7,155,819 raw sequencing reads were produced per sample, and 42,771,617 ± 6,452,084 reads per sample were retained after quality control with Trimmomatic tool v.0.38. (read ends clipped if average quality score was <20 within the 5 bp sliding window) and human genome sequence decontamination with Bowtie2 v.2.3.5.1 and SAMtools v.1.7 using the GRCh38 reference genome. A taxonomic identity assignment of the filtered reads ≥ 100 bp in length was performed with the Kraken2 v.2.0.8-beta classifier using the RefSeq database examining 35 bp *k*-mers. Only reads achieving a Kraken2 classification confidence score of 0.5 or higher were considered successfully classified and processed with Bracken v.2.5. for an estimation of the relative abundance of each species and a re-estimation of abundances at higher taxonomic levels (e.g., summing up reads representing the same genus).

**Statistical analysis.** The median of 1000 reads was used as a cut-off value for taxa to be retained for statistical analysis in the RStudio environment. The alpha diversity of the gut microbiome was characterized using the Shannon index and the effective number of species, estimated as the exponential of the Shannon index using read counts [46]. The within-subject correlation coefficients (CV_ws_) were calculated using the “root mean square approach”. To account for sequencing depth differences among the samples, before continuing with the statistical analysis of the compositional data, the read counts for the taxa were normalized using centred log-ratio transformation. Sample distribution regarding the microbiome composition and lipid profiles was characterized using principal component analysis (PCA), calculated using the R “stats v.4.0.2” *prcomp* function, and biplots were created using the package “factoextra v.1.0.7” [47]. The lipid measurements were calculated as z-scores before PCA for normalization, and “high”/”low” categories were defined based on reference thresholds for the healthy lipid range: TCh < 5.0 (mmol/L), HDL ≥ 1.2 (mmol/L), non-HDL Ch ≤ 3.9 (mmol/L), LDL < 3.0 (mmol/L), and TG < 1.7 (mmol/L). Differences in the beta diversity between the sample categories were assessed with PCA and the *adonis* function in the package “vegan v.2.5-7” (999 permutations, strata = “subject” for repeated measures). The proportion of variation in the relative abundance of taxa explained by the different variables was estimated with the package “variancePartition v.1.20.0” [48]. Both the PCA and variation partitioning analysis were completed using follow-up data, which included all eight samples collected from each participant. Correlations between the taxa relative abundances and the follow-up serum lipid measurements were calculated with packages accepting repeated measures “rmcorr v.0.4.1” (repeated measures correlation) and “MaAsLin2 v.1.4.0” (Microbiome Multivariable Associations with Linear Models 2), which also allows for covariate adjustments through a mixed-effect model [49,50].

The average relative abundances of the taxa between the two categories defined by “high”/”low” lipid levels were compared using the Wilcoxon rank sum test in “rstatix v.0.7.2” and “MaAsLin2 v.1.4.0“. The Wilcoxon rank sum test was also used to assess the differences in age, BMI, and average serum lipid measures between men and women. The Wilcoxon signed-rank test in “rstatix v.0.7.2” was employed for paired samples to assess the potential impact of the consumption of target products on the taxa relative abundances and serum lipid levels. The test compared samples collected before and after each intervention week. Correlations between the average taxa relative abundances and phenotypes were calculated using Spearman correlation in “Hmisc v.4.4-2”, “rstatix v.0.7.2”. Confidence intervals (CI 95%) for Spearman’s rho were estimated in “DescTools v.0.99.50“using function “SpearmanRho”. Kendall rank correlation was used to compare the ordinal data with continuous variables in “rstatix v.0.7.2” in order to estimate correlations between the habitual diet-related factors, and average the relative abundance of the taxa and mean serum lipid levels. *p*-values were corrected for multiple testing using the Benjamini–Hochberg method. The 95% prediction intervals were calculated following the formula:

y_0_ ± t_α/2,df_ = n − 2 ∗ SE (1)

where standard error (SE) is:

(2) $${\mathrm{SE}=\mathrm{SE}}_{\mathrm{yx}}\sqrt{\left(1+1/n+{\left(x_{0}\;-\;x_{\mathrm{av}}\right)}^{2}{/\mathrm{SS}}_{x}\right)}$$

The “y_0_” is the forecasted value for “x_0_”, “x_0_” is the median value of the relative abundance of the taxon, “x_av_” is the average relative abundance of the taxon, “t” is a critical value of α/2 (α = 0.05), “df” is the degree of freedom calculated as “n − 2”, where “n” is the number of subjects, “SE_yx_” is the SE of given x and y values and “SS_x_” sum of squares of deviations of data points “x_av_”. Prediction intervals were calculated in “MS excel” using average values of variables extrapolated from longitudinal data.

Graphs were created with the “ComplexHeatmap v.2.7.4”, “ggplot2 v.3.4.2”, “ggpubr v.0.6.0”, and “factoextra v.1.0.7” packages.

## 3. Results

### 3.1. Participant Description

From the 23 participants, 7 men and 14 women completed all eight visits, and two women completed seven and five visits, respectively. The total data pool contained 180 samples. The participants had a mean BMI slightly above the recommended range (26.3 ± 5.01 kg/m^2^) and were aged between 27 and 62 years, with the women being, on average, six years older than the men (Table 1). Among all study participants, 13 had increased mean circulating total Ch levels (≥5.0 mmol/L). The average circulating LDL levels were above the recommended threshold (<3.0 mmol/L) for 14 participants, but only four participants (two men and two women) had increased serum TG levels (≥1.7 mmol/L). The women had, on average, higher total Ch levels than the men. The levels of LDL, non-HDL Ch, and HDL were also higher among the women, but the differences were not significant. In contrast, the average TG levels tended to be slightly higher in the men than the women (Table 1). The individual-specific serum cholesterol parameters remained relatively stable over the study period (from CV_ws_ 6.3% for HDL to CV_ws_ 11.1% for LDL), but there was notable variation in the circulating levels of TG (CV_ws_ 27.8%; Figure S1).

When taxa with a median number of reads of less than 1000 were removed, 24 families, 40 genera, and 69 species of bacteria and archaea remained to investigate the relationships between the lipid profiles and the gut microbiome composition of the participants. The average inner or alpha diversity of the gut microbiome of the participants is expressed as the effective number of species, which was 20.82 ± 4.41, and the average Shannon’s diversity index was 3.01 ± 0.22. The variation in alpha diversity over the study period was moderate, with CV_ws_ for an effective number of species of 13.8% and 4.8% for Shannon’s diversity index (Figure S2). Participant-related cofactors like gender, BMI, and age showed no correlation with either the effective number of species or Shannon’s index (Figure S3). The participants with high serum HDL levels (HDL ≥ 1.2 mmol/L) tended to have more diverse microbial communities according to Shannon’s index (3.06 ± 0.144 vs. 2.86 ± 0.24), although the differences were not significant after multiple testing corrections (*p* = 0.046 vs. *p*_adj_ > 0.05; Table S1). There were no differences in the average alpha diversity between participants with hypercholesterolaemia or increased TG levels (Tables S1 and S2; Figure S4). Likewise, Spearman’s correlation and repeated measures correlation showed no association between the alpha diversity and average lipid measurements of the participants or the measurements taken at each visit (Table S1 and Figure S5).

**Figure 1:**
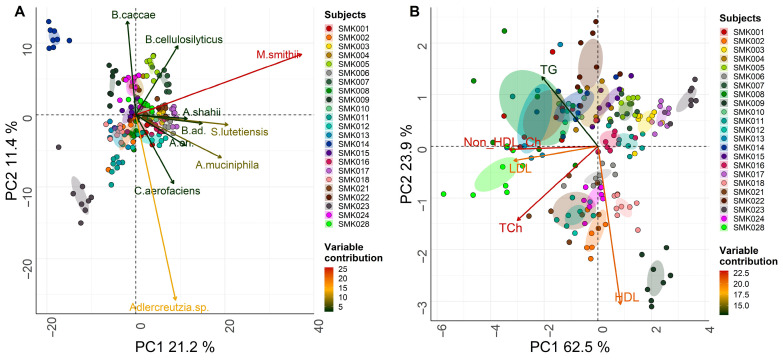
The PCA plot showing the clustering patterns of the samples. Sample distribution patterns based on (**A**) participant-specific gut microbiome profiles and (**B**) on participant-specific lipid profiles. Arrows indicate the top ten contributing bacterial species and lipid variables. The arrows’ colour corresponds to each variable’s contribution to the principal axes. Ellipses show 95% confidence intervals. Dots are coloured by participant. The first two principal components, PC1 and PC2, explained the highest proportion of variation. A.on.—*A. onderdonkii*, B.ad.— *B. adolescentis*, TCh—total cholesterol, HDL—high-density lipoprotein, LDL—low-density lipoprotein, and TG—triglyceride.

As expected, a visual examination of the data separation using PCA of the gut microbiome composition at the species level revealed a sample clustering for the participants in agreement with the well-known fact that the gut microbiome is highly individual-specific (proportions of variation explained by principal components (PC) PC1 and PC2: 21.2% and 11.4%, respectively; Figure S6). The top contributing species explaining the variability among the samples were *Methanobrevibacter smithii*, *Streptococcus lutetiensis*, *Akkermansia muciniphila*, and *Bifidobacterium adolescentis*, which contributed the most to PC1 (37.8%, 11.7%, 10.0%, and 6.05%, respectively), while *Adlercreutzia* sp. *8CFCBH1* and *Bacteroides caccae* contributed 33.6% and 8.6% to PC2, respectively (Figure 1 and Table S3). At higher taxonomic levels, the majority of variation among subjects was explained by the genera *Methanobrevibacter* (contributing most to PC1: 60.7%) and *Adlercreutzia* (contributing most to PC2: 59.5%), and the families *Methanobacteriaceae* and *Akkermansiaceae* (contributing 76.7% and 72.1% to PC1 and PC2, respectively; Table S3, Figures S6 and S7). Although the first two PCs in the PCA based on the lipid profiles explained a higher proportion of variance than the microbiome data (PC1: 23.9%; PC2: 62.5%), the subject-specific clustering of samples was less pronounced than the microbiome-based patterns. The strongest contributor to PC1 among the lipid parameters was non-HDL Ch (31.5%), followed by LDL (28.2%) and total Ch (25.9%), whereas HDL was the most prominent contributor to PC2 (70.4%; Figure 1 and Figure S8).

**Figure 2:**
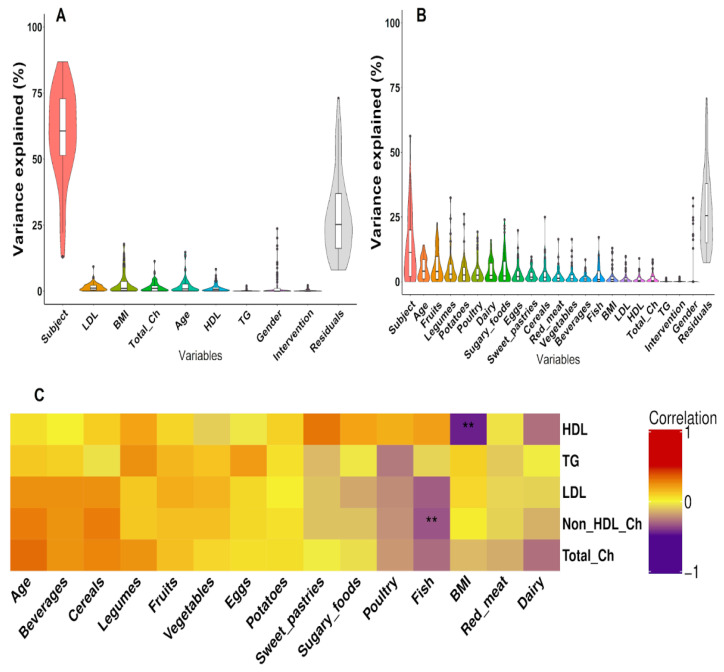
Correlation of participant-related covariates with microbiome composition variance and lipid profiles. (**A**) Variation in gut microbiome composition attributable to lipid parameters and major confounders. (**B**) The variance proportion explained by the subject-related factors, including habitual diet. The non-HDL Ch measurements were excluded from both models due to the high correlation rate with other lipid parameters. The collinearity scores were 0.87 and 0.91 for the first and the second model, respectively. The residual group comprises undefined contributors to the variance. Variance partitioning included a complete set of samples for each participant. (**C**) Heatmap showing a correlation between average lipid measurements and participant-related variables. **—unadjusted *p*-values < 0.05. Total Ch—total cholesterol, HDL—high-density lipoprotein, LDL—low-density lipoprotein, TG—triglycerides, and BMI—body mass index.

In concordance with the PCA analysis, variance partitioning supported the host itself being the most potent factor shaping the composition of the gut microbiome, with an average of 59.3% of the species level variance explained. The average variation explained by the summary effect of lipid variables was 4.5% in the longitudinal data set. The contribution of cholesterol-related parameters ranged from 1.6% for total Ch to 1.2% for HDL, whereas the TG levels explained a negligible 0.2% of the total bacterial variance. The intervention diet also had a low impact, explaining 0.3% of the total variation in the structure of the gut microbiome. On the contrary, the habitual diet together explained, on average, 43.6% of the variance in the relative species abundance, with the highest proportion of variation related to the consumption of fruits (proportion of variation explained: 4.8%). However, a substantial proportion of variance was attributable to factors unaccounted for within the analysed models (Figure 2). The variables included in the variance partitioning of the microbiome composition showed similar contribution patterns at the genus and family levels (Figure S9).

### 3.2. Interaction of Lipids, Bacteria, and Individual Characteristics

In general, the participant-related factors had no significant impact on the average lipid levels of the participants. The only variables that marginally correlated with HDL and non-HDL Ch levels were BMI and inclusion of fish products in the habitual diet (Spearman’s rho = −0.46, *p*-value = 1.40 × 10^−6^, *p*_adj._ > 0.05; Kendall’s tau = −0.35, *p*-value = 0.041, *p*_adj._ > 0.05). The serum total Ch tended to increase with age, though the correlation was non-significant (Figure 2).

A PCA analysis was performed with the lipid measurements as categorical variables to explore whether variation in the compositional structure of the gut microbiome is sufficiently pronounced in the group participants based on their average blood lipid levels. For this analysis, the standard fasting cut-off values defined for serum lipids in routine testing were used to ascribe participants to the “high” or “low” lipid group according to their average serum lipid measurements. Although there was some notion of separation between groups with “high” or “low” levels of blood HDL based on their gut microbiome profile, it was not strong enough to indicate significant differences in gut microbiome composition between the two groups. The tested grouping patterns explained 7% of the variance at the species level (*p* = 0.02), 10% at the genus level (*p* = 0.07), and 10% at the family level (*p* = 0.05). There was no evidence of microbiome-based clustering of participants with differences in other lipid-related measurements (Figure S10).

**Figure 3:**
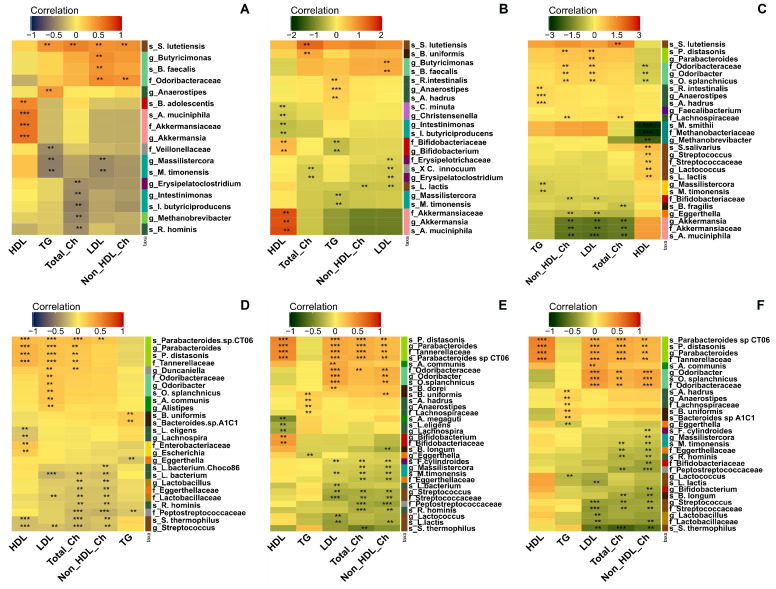
Correlation between measurements of serum lipids and relative abundance of microbial taxa representing gut microbiome. Correlations were estimated using cross-sectional data with (**A**) Spearman correlation followed by Maaslin2’s linear models, (**B**) unadjusted and (**C**) adjusted for cofactors age, BMI, and gender. For estimates based on the follow-up data set, analysis methods included (**D**) repeated measures correlation followed by Maaslin2’s linear models, (**E**) unadjusted and (**F**) adjusted for cofactors. The colours of the cells correspond to Kendall’s tau, and asterisks indicate *p*-values. The colours of the annotation bar show relatedness between taxa. **—*p* < 0.05; ***—*p* < 0.01; s—species; g—genus; f—family.

Association patterns between the bacterial taxa and lipid profiles were described in two steps. The first step was to estimate correlation patterns using the follow-up data from the eight study visits. The second step included correlation analyses based on the average lipid measurements and average relative abundances of taxa calculated for each participant from the follow-up data. A total of 29 species, 20 genera, and 13 families showed a marginal correlation with circulating lipid levels. However, none of these associations retained significance after correction for multiple tests, and the described results are based on raw *p*-values. The association results are presented in Figure 3 and Table S4.

Several taxa showed consistent correlation patterns across both the cross-sectional and follow-up data sets. The relative abundance of *Odoribacter splanchnicus* from the *Odoribacteraceae* family positively correlated with serum LDL and non-HDL Ch levels (Table S4). The tendency of *O. splanchnicus* to be more common among participants with high LDL measurements was supported by several tests. The species *O. splanchnicus* was positively correlated with sample-to-sample LDL measurements (r_rm_ = 0.197, Figure 3D; coef = 0.351, Figure 3E) and tended to be more abundant among participants with higher average levels of LDL (Figure 3C). These correlations retained significance after adjustments for age, gender, and BMI (follow-up data coef_adjust_ = 0.429, Figure 3C; cross-sectional data coef_adjust_ = 0.388, Figure 3F). Two species, *Parabacteroides distasonis* and *Parabacteroides* sp. *CT06,* also tended to be increasingly abundant with higher serum cholesterol levels, including HDL (Table S4). The association between the abundance of *P. distasonis* and increased circulating cholesterol was consistent across both data sets (Figure 3C–F), whereas *Parabacteroides* sp. *CT06* correlated with fluctuations in serum cholesterol levels in the follow-up data (Figure 3D,F). The correlations between LDL and *P. distasonis* remained significant after including cofactors in the association analysis using follow-up and average measurements (coef_adjust_ = 0.365 and coef_adjust_ = 0.652, respectively, Figure 3C and Figure 3F).

Negative correlations with the serum total Ch, LDL, and non-HDL Ch levels were observed for species from the genera *Streptococcus* and *Lactococcus,* which belong to the *Streptococcaceae* family (Table S4). *Lactococcus lactis* was negatively associated with the follow-up and average measurements of LDL and non-HDL Ch in serum. A negative association with sample-to-sample fluctuations in LDL levels was retained after adjusting for age, BMI, and gender in the analysis (coef_adjust_ = −0.363; Figure 3F). After cofactor adjustment, a positive correlation was revealed between the average abundance of *L. lactis* and serum HDL (coef_adjust_ = 0.544; Figure 3C). The abundance of *Streptococcus thermophilus* showed a negative correlation with sample-to-sample fluctuations in cholesterol levels in the follow-up data set (Figure 3D–F). The adjusted correlation remained significant (coef_adjust_ = −0.717 with total Ch levels), but we cannot exclude the impact of cofactors since *S. thermophilus* was more abundant among older participants (linear model coef = 0.65; Table S5). *S. lutetiensis* was positively associated with average serum lipid levels, including TG (Figure 3A–C).

The species *Bifidobacterium longum* and *B. adolescentis* were associated with healthier lipid profiles (Table S4). *B. longum* negatively correlated with non-HDL Ch levels (coef_adjust_ = −0.383; Figure 3F), and *B. adolescentis* positively correlated with average HDL levels (Spearman’s rho = 0.495; Figure 3A). The participants with a lower BMI had a significantly higher *B. adolescentis* abundance, which may have affected the association patterns (Table S5). The recently classified species *Massilistercora timonensis* was relatively enriched in samples with lower cholesterol levels, particularly non-HDL Ch (coef = −0.224 and coef_adjust_ = −0.214; Figure 3E,F). Furthermore, the species was more abundant among participants characterized by lower average serum TG levels, independently of cofactors (coef_adjust_ = −0.490; Figure 3A–C and Table S4).

The participants with lower average circulating cholesterol levels had a higher relative abundance of probiotic *A. muciniphila*. This species positively correlated with average serum HDL levels (Spearman’s rho = 0.599; Figure 3A,B). Yet, when participants’ age, BMI, and gender were taken into consideration, the abundance of *A. muciniphila* negatively correlated with total and LDL cholesterol levels in serum (Figure 3C and Table S4).

Species from the family *Lachnospiraceae*, including *Roseburia hominis*, *Lachnospira eligens*, *Lachnospiraceae bacterium*, and *Lachnospiraceae bacterium Choco86,* correlated with decreased levels of circulating cholesterol. The well-known butyrate-producer *R. hominis* was associated with lower levels of total circulating cholesterol and non-HDL Ch in the follow-up data set, independently of the participants’ age, BMI, or gender (coef_adjust_ = −0.290 and coef_adjust_ = −0.267, respectively; Figure 3F and Table S4). However, the negative associations with total Ch, non-HDL Ch, and HDL observed for *L. eligens*, *L. bacterium,* and *L. bacterium Choco86* lost significance after adjustment for participant-specific cofactors, particularly gender (Figure 3D–F and Table S4). These species tended to be more abundant among men, who were characterized by lower serum cholesterol levels than women, with significant differences observed for *L. bacterium* (Table S5). Likewise, adjustments for cofactors, predominantly gender, removed the correlation between lipid levels and the relative abundance of *Roseburia intestinalis*, *Intestinimonas butyriciproducens*, *Christensenella minuta*, and *Bacteroides dorei* (Figure 3 and Table S4). The abundances of these species differed significantly between men and women (Table S5).

Apart from the described species, the taxa associated with unfavourable serum cholesterol levels were the genus *Duncaniella* and several species, including *Alistipes communis*, *Alistipes megaguti*, and *Butyricimonas faecalis*. The taxa that inversely correlated with circulating lipids included the family *Peptostreptococcaceae*, the genus *Escherichia*, and the species *M. smithii* and *Bacteroides fragilis* (Table S4).

In general, there were fewer associations with TG levels in the blood. *Anaerostipes hadrus* had the most consistent positive correlation with TG levels. The model based on the average measurements adjusted for cofactors showed the strongest association for this species (coef_adjust_ = 0.284; Figure 3F). *Bacteroides uniformis* positively correlated with short-term changes in serum TG levels (Figure 3D–F). Although adjustment for cofactors did not remove the association, the abundance of *B. uniformis* differed between genders, which could have affected the results (Table S5). Besides a negative correlation with cholesterol levels, the genus *Eggerthella* was also inversely correlated with TG levels (Figure 3B–F).

### 3.3. Impact of Diet

Diet is a potent modulator of the gut microbiome and can affect serum lipid profiles. Therefore, we also checked for potential confounding effects of habitual diets and the introduction of additional meat products on the correlations between the microbial communities and circulating levels of lipids. As described previously, consuming more fish and sea products lowered serum cholesterol levels, particularly serum non-HDL Ch (Kendall’s tau −0.22, *p*-value = 0.041; Figure 2). The daily consumption of salmon as a part of the dietary intervention resulted in mildly lower levels of serum TG and LDL (average TG levels of 1.30 ± 0.83 mmol/L and LDL levels of 3.01 ± 0.82 mmol/L in reference to 1.05 ± 0.46 mmol/L and 2.85 ± 0.79 mmol/L in samples after intervention, *p* < 0.05; Figure S11).

Habitual diet explained a substantial proportion of the participants’ total gut microbial community composition variation. However, fish and sea products were not among the top contributors (proportion of variation explained: 1.5%; Figure 2). However, the Kendall correlation analysis indicated that consuming fish products may have affected the relative abundance of several taxa that were previously linked with variations in serum lipid levels. Among these were *L. bacterium*, *S. salivarius*, *A. muciniphila,* and bacteria from the genus *Lactobacillus* (Figure S12). A more frequent consumption of dairy products correlated with a higher relative abundance of *C. minuta* and *I. butyriciproducens*, whereas *M. timonensis* showed an inverse correlation with dietary fruits and legumes. Species from the *Bacteroidaceae* family tended to be more abundant among subjects preferring sugary foods (Figure S12).

An initial visual check for data clustering with PCA comparing the pre- and post-intervention samples showed no intervention-related patterns in the gut microbiome (maximum Adonis R^2^ = 0.011), which is in line with the weak effects revealed by the variation partitioning analysis (Figure 2 and Figure S13). Together, these results indicate that the consumption of the three intervention food products did not have notable effects on the general taxonomic structure of microbial communities. Variations in the relative abundance of individual microbial taxa in response to the consumption of fish or meat were assessed with Maaslin2, including pre- and post-intervention samples for each study product with the subject as a random effect grouping parameter. The consumption of the intervention food products affected the relative abundances of six lipid-associated species, *R. intestinalis*, *R. hominis*, *L. bacterium Choco86*, *C. minuta,* and *B. adolescentis*, as well as the genera *Faecalibacterium,* and *Bacteroides*, and the families *Peptostreptococcaceae*, *Eggerthellacea*, and *Akkermansiaceae* (Figure S14).

**Figure 4:**
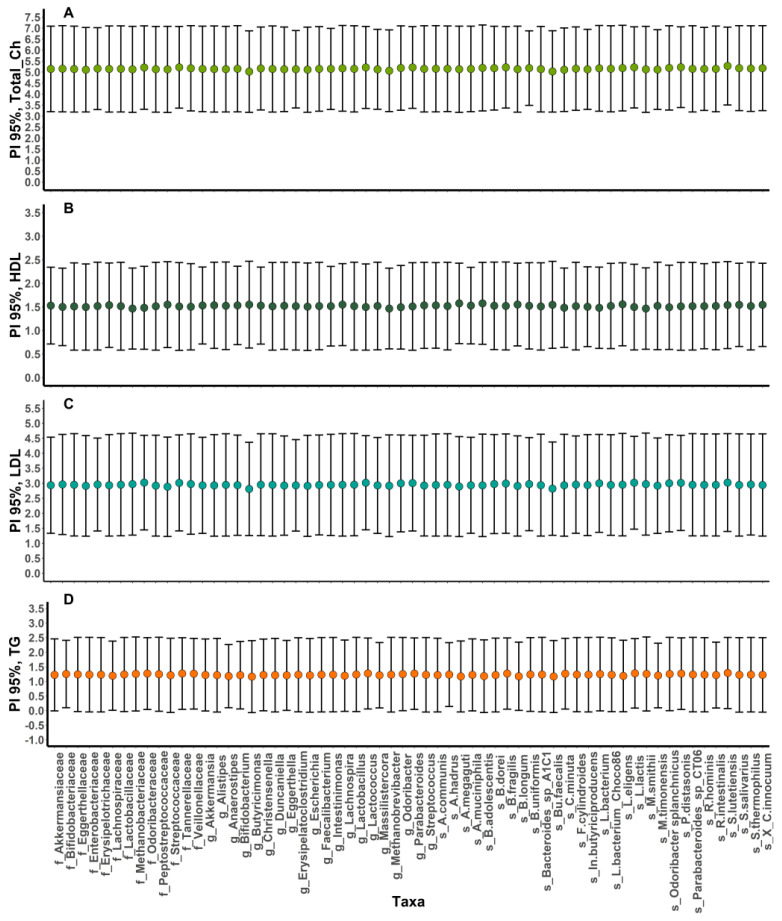
Prediction intervals characterizing the uncertainty of blood lipid level forecasts based on the relative abundance of bacterial taxa. Plots (**A**–**D**) display 95% prediction intervals estimated for total Ch, HDL, LDL, and TG levels in blood, respectively. The whiskers indicate the intervals within which true lipid measurement values corresponding to the respective taxon’s relative abundance will be found with 95% confidence. The point indicates the lipid level forecasted for the median relative abundance of the respective taxon. Prediction intervals for non-HDL Ch levels exhibited similar patterns to those observed for LDL and, therefore, are not included in this plot (Figure S4). PI—95% prediction interval.

However, the strength of the observed correlations was generally relatively low, and the data exhibited high variability. Therefore, to indirectly address the uncertainty of these observed correlations, prediction intervals were calculated for the blood lipid levels forecasted based on the relative abundances of taxa associated with lipid parameters (Figure 4 and Table S4). The 95% prediction intervals were characterized by a high bandwidth and relatively homogeneous patterns among the taxa. This supports the idea that, apart from other factors, at least some of the associations could be modulated by confounders, such as gender.

## 4. Discussion

The current short-term longitudinal study describes the correlation between gut microbiome composition and blood lipid patterns in healthy adults. The investigation using a data pool comprising gut microbiome taxonomic profiles and lipid measurements collected from 23 participants confirmed that features of the human gut microbiome vary with subject-specific lipid profiles.

### 4.1. Characterization of Gut Microbiome Profiles

Circulating levels of lipids are determined by numerous host-related and environmental factors like genetics, gender, smoking status, BMI, physical activity, and diet [3,51]. More recently, the multifunctional gut microbiome ecosystem has been described as one of the critical regulators of complex metabolic networks that maintain host lipid turnover [8,11,12,13,14]. A high overall richness of the gut ecosystem is typically associated with improved metabolic health, whereas patients with obesity, type 2 diabetes, or metabolic syndrome have reduced bacterial diversity in their gut communities [5,14,52]. Similar trends have been found in relation to circulating lipid levels. Patients with hyperlipidaemia have been described as having less diverse microbiomes compared to normolipidaemic controls [5,14,52]. Individuals with healthier blood lipid profiles, namely lower LDL and TG levels and higher HDL levels, also had richer gut microbial communities [8,12,14,17]. However, not all studies agree on such correlations [9,15,36]. One of the explanations could be that the composition of the community and the presence of particular members such as SCFA producers may be functionally more relevant than the overall diversity of the gut microbiome [36]. We observed no significant relationships between indices describing microbial diversity and lipid levels among healthy participants with normal or moderately increased blood lipid levels. Nevertheless, participants with higher average HDL levels also tended to have a more diverse microbiome than those characterized by lower average circulating HDL levels, which aligns with observations in population-based Dutch cohorts [8,12].

The study in the Dutch cohort by Fu et al. [8] revealed that approximately 6% of the variance in circulating lipid levels could be attributed to the gut microbiome composition, independent of major confounders like age, gender, and host genetic factors. The gut microbiome explained approximately 4% of the variation in HDL and 6% in TG levels, with a much lower proportion accounting for total Ch and LDL levels (0.7% and 1.5%, respectively) [8]. A variance partition analysis yielded similar results within our small study cohort, with an average of 7% variance in the gut microbiome related to lipid levels. However, we saw weaker relationships between HDL and TG levels and the gut microbiome composition. There was a high percentage of variance in the gut microbiome that was explained by unknown factors and by subject-specific factors other than age, gender, and BMI, and even habitual diet-related components. Such confounders left unaccounted for could have affected our estimates. Differences in the estimated contribution to the variance in the gut microbiome could be due to genetic factors, population-specific lifestyle-related factors like diet, or distinct features of the gut microbiome itself [6,8,53].

Subjects with hyperlipidaemia can be differentiated from the normolipidaemic population based on the composition of their gut microbiome communities [5,13,14,52]. We observed some clustering between participants assigned to “low” or “high” serum HDL groups. Otherwise, there were no significant differences in the general patterns of gut microbial core communities between participants with high or low circulating lipid measurements. Within the small cohort, substantial subject-specific variation might have obscured sample clustering between groups with “high” and “low” levels of lipid variables. The total Ch levels were, on average, only borderline-high among participants within the “high” lipid group. Therefore, cases were probably more similar to controls in terms of the gut microbiome than patients diagnosed with hyperlipidaemia would be [5]. However, other studies involving individuals with hyperlipidaemia also found no notable differences in the overall taxonomic structure of the gut microbial communities between cases and controls [9,15,17]. A dietary intervention study investigating the effects of saturated fats and proteins on the human gut microbiome indicated that a high alpha diversity correlated with a more stable and impact-resistant gut microbial community structure [54]. Two of the three studies in which no segregation based on microbiome structures was observed between cases and controls also found no significant differences in alpha diversity between hyperlipidaemic and normolipidaemic individuals [9,15].

### 4.2. Taxa Associated with Serum Lipid Levels

Although no relationships were observed between the general composition of the gut microbiome and circulating lipids, the relative abundances of several taxa correlated with serum cholesterol and triglyceride levels. Most associations were detected at the species level, and correlations at the genus and family levels mainly reflected those of specific species.

Among the taxa associated with lipid levels were bacterial species capable of SCFA production. SCFAs are major metabolites of the gut microbiome, produced from the fermentation of dietary fibre and other non-digestible polysaccharides [31,35]. A high rate of SCFA production in the intestines is generally considered as health-promoting [31]. Microbial SCFAs interact with the host metabolism by lowering food intake, metabolizing and promoting lipid excretion, and regulating lipogenesis, cholesterogenesis, and gluconeogenesis [32,33,34,35]. Moreover, SCFAs can improve the function of the gut barrier, reduce local inflammation, as well as interact with the immune system on the organism level, which indirectly contributes to the host’s metabolic health [35].

SCFA producers correlating with lipid levels in the current analysis were species from the genera *Alistipes*, *Anaeorostipes*, *Bifidobacterium*, *Butyricimonas*, *Intestimonas*, *Roseburia*, *Odoribacter*, *Lachnospira*, and unclassified *Lachnospiraceae*. The representatives of these genera have been linked with host metabolic traits, like obesity and hypercholesterolaemia, in previous studies, but with inconsistent results [6,11,13,35,55,56,57]. For the majority of SCFA producers, we observed positive correlations with healthy lipid profiles characterized by lower serum LDL, non-HDL-Ch, and TG levels. A higher abundance of the *Lachnospiraceae* family has been consistently linked with improved lipid profiles and lower obesity-related markers in several studies among healthy adults [11,13,55,56,57]. Gargari et al. observed improvements in serum lipid profiles and SCFA levels among hyperlipidaemic children and adolescents after eight weeks of hazelnut intake. The beneficial changes in metabolic features were accompanied by an increased abundance of SCFA producers including *Lachnospiraceae*, particularly *Roseburia* [52]. Apart from butyrate, *Roseburia* produce conjugated linoleic fatty acid, which also contributes to lipid turnover regulation [6]. Similarly, *Bifidobacterium* has been correlated with lower plasma LDL and TG levels [3,13], which is in line with our findings. The administration of *Bifidobacterium*-based probiotics significantly reduces serum cholesterol and triglyceride levels in human cohorts [6,31,58]. However, the abundance of the *Bifidobacterium* genus was higher among Japanese patients with hyperlipidaemia and type 2 diabetes [17]. Members of this genus also produce lactate and acetate in addition to butyrate, and modify the bile acid pool [59]. Some *Bifidobacterium* species can also directly interact with dietary cholesterol and produce cholesterol sulphate, although the role of this metabolite in the context of hyperlipidaemia is unknown [42].

The species *O. splanchnicus* and *A. hadrus* were more abundant in samples with higher LDL, non-HDL Ch, and TG levels. The representatives of the genus *Odoribacter* were enriched among subjects with abnormal lipid profiles compared to normolipidaemic ones in other studies [5,11]. A higher *Odoribacter* abundance among hypercholesteraemic men was accompanied with increased levels of isobutyric acid compared to controls [5]. Isobutyric acid levels were associated with increased serum lipid levels among pregnant women [39]. The gut microbiome enrichment in *Anaerostipes* has been related to an increased risk of obesity in children which corresponds with *A. hadrus* being more abundant among participants with higher TG levels in our study [12,42]. However, a study including over 800 participants described a strong negative correlation between the relative abundance of the genus *Anaerostipes* and blood TG levels [11]. Of note, *Anaerostipes* was negatively correlated with LDL levels among men, but not among women in a Japanese cohort [9]. Both of the species *O. splanchnicus* and *A. hadrus* produce acetate and propionate [60]. Theoretically, switching towards a higher production of acetate could explain the increased lipogenesis and cholesterogenesis, resulting in higher serum lipid levels [35,61]. *Odoribacter* along with *Alistipes* are among the few taxa capable of producing sulfonolipids, which may be involved in triggering inflammation [62]. *Anaerostipes* utilizes lactate and succinate and could co-occur with taxa producing other SCFAs [63]. However, causal mechanisms linking both species with the regulation of serum lipid levels remain obscure.

We were able to confirm positive associations with favourable serum lipid compositions for *A. muciniphila* and *C. minuta*. Both species have been considered as next-generation probiotics for the treatment of metabolic diseases [31,64]. Mucin degrader *A. muciniphila* is intensively researched due to its health-improving effects, including reduced adiposity, improved glucose metabolism, improved gut barrier integrity, and anti-inflammatory and anti-ageing effects [65]. This species also participates in the regulation of the host’s cholesterol metabolism. Healthy individuals with a higher abundance of *Akkermansia* have been observed to have healthier circulating lipid profiles, while the genus was found to be depleted in hyperlipidaemic patients [8,52,56]. A study involving dyslipidaemic patients suggested a potentially causal relationship between *Akkermansia* and abnormal lipid levels among women, but not in male participants [9]. When used as probiotic supplement, *Akkermansia* decreased fat mass and reduced total Ch, LDL, and TG levels in the serum of obese volunteers [66]. The regulation of lipid turnover in the liver is partly mediated by its metabolites, acetate and propionate [65]. A recent study demonstrated that *A. muciniphila* can inhibit cholesterol synthesis in the colon through mechanisms involving the mucin utilization locus (MUL) gene complex [67].

Bacteria from the *Christensenellaceae* family are typically associated with lean phenotypes [8,31,64]. *Christensenellaceae* have been described as more abundant among individuals with favourable serum lipid profiles, characterized by low total Ch, LDL, and TG levels and high HDL [8,13,64,68]. *C. minuta*’s anti-obesity effects are presumably mediated by the inhibition of hepatic lipogenesis and the strengthening of the gut epithelial barrier. This species inhibits hepatic glucokinase, a regulator of glycolysis and lipogenesis [69]. *C. minuta* also produces acetate and butyrate, participates in secondary bile acid synthesis, and has shown cholesterol-reducing properties, at least in cultures [31,69,70].

Bacteria correlated with healthy serum lipid profiles, including higher HDL levels and lower total Ch and LDL, also included other species with probiotic potential, representing the genera *Streptococcus*, *Lactococcus*, and *Lactobacillus*. The capability of probiotic bacteria to alleviate hyperlipidaemia has been supported by studies investigating the effects of probiotic supplementation among patients with obesity and mild-to-high hyperlipidaemia [19,20,58]. The suggested mechanisms mediating the cholesterol-lowering effects of probiotics include SCFA production, bile acid modulation, and cholesterol transport and metabolism [31,32,71]. Species of *Lactobacillus* and *Streptococcus* have been also demonstrated to be able to directly bind cholesterol [72].

The probiotics *L. lactis* and *S. thermophilus* are considered to be food-borne and therefore transient species in the gut microbiome [73,74]. The administration of both probiotics had beneficial effects on circulating lipid profiles in animal models, supporting their protective role against dyslipidaemia [73,75]. On the contrary, *S. lutetiensis* is a common resident species of the human gut. Bacteria from *Streptococcaceae* were enriched in a murine model of obesity after a high-fat diet treatment [69]. It was also found to be more abundant among women with dyslipidaemia and among obese adults [9,76]. Adolescents with obesity also were characterized by a higher abundance of *Streptococcaceae*, identified as *S. thermophilus* and *S. salivarius* [77]. Species from the genus *Streptococcus*, particularly *S. thermophilus* and *S. salivarius*, can be misidentified due to high sequence similarity [74]. Therefore, to distinguish between the metabolic effects of different *Streptococcus* species, more studies with controlled dietary probiotic intake are necessary.

Bacteria from the genus *Parabacteroides* are known to be engaged in cholesterol metabolism and to regulate other obesity-related traits [78]. *P. distasonis* and *Parabacteroides sp CT06* were correlated with higher blood LDL, HDL, and non-HDL Ch levels, and, subsequently, total Ch measures in the study cohort. This contradicts the overall beneficial impact on metabolism attributed to *Parabacteroides* species. The administration of *P. distasonis* ameliorated obesity and adverse lipid profiles in a murine model. The species produces acetate and succinate. Succinate interacts with the gastrointestinal nervous system to reduce food intake and regulate glucose homeostasis. *P. distasonis* also upregulates the production of secondary bile acids, triggering signalling cascades downstream of FXR and TGR5, thus modifying gluconeogenesis and lipogenesis [31,78]. Although there is some evidence that decreasing *Parabacteroides* abundance might contribute to the cholesterol-lowering action of metformin and certain prebiotics, the mechanisms underlying these findings are not clear [79,80].

We also detected correlations with serum lipid levels for other taxa comprising bile acid-metabolising members. *Alistipes* species have been described as animal-based, protein- and fat-rich diet-favouring, bile-acid tolerant bacteria [38,81]. Gut microbial communities of mice receiving gut microbiome transplants from patients with increased serum cholesterol were characterized by an increased abundance of *Alistipes*, indicating a positive link with host circulating lipid levels. *Alistipes* participates in bile acid metabolism, and the synthesis of sulfonolipids and SCFAs, predominantly acetate [62,82]. Another mechanism linking *Alistipes* with aberrant lipid levels could be the production of lipopodenglysaccharide (LPS) [81]. However, Takagi and colleagues found that *Alistipes* was significantly depleted among hypercholesterolaemic individuals [17].

Members of the *Peptostreptococcacea* family, in line with the observed correlation patterns, are known for their ability to modify bile acids and reduce of cholesterol levels by converting it to coprostanol [32]. *Peptostreptococcus* has been previously correlated with metabolic diseases like type 2 diabetes and metabolic syndrome [83,84]. Apart from *Peptostreptococcaceae*, *M. timonensis*, another representative of the *Eubacteriales* order within *Firmicutes* phylum, was associated with decreased total Ch and LDL levels, showing moderate but consistent effects. *M. timonensis* is a recently described and poorly characterized species related to bacteria from the *Faecalicatena* genus from the *Lachnospiraceae* family [85]. The administration of plant-derived bioactive phenylethanoid glycosides increased the abundance of *M. timonensis*, concurrently decreasing total Ch, LDL, and HDL levels in a mouse model of obesity, suggesting a possible link between the species and circulating lipids [86]. In the current study, participants who reported a higher consumption of fibre-rich foods such as fruits and legumes had a low relative abundance of this species. It is possible that, similar to closely related species from the *Faecalicatena* genus, *M. timonensis* favours an animal-based, high-fat diet [87]. Together, this indicates that *M. timonensis* may modulate the host’s lipid metabolism, but the species’ role in the human gut microbiome requires further investigation.

Unrelated taxa with association signals detected through a single test included the families *Veillonelacea* and *Erysipelotrichacea*, the genera *Escherichia*, *Eggerthella, Bacteroides*, and *Duncaniella*, and the species *M. smithii* and *Faecalitalea cylindroides*. Thus far, the family *Erysipelotrichaceae* and its member *F. cylindroides* have been associated with hypercholesterolaemia, although the metabolic pathways linking *Erysipelotrichaceae* with aberrant lipid metabolism have not been clearly indicated [29,88]. A positive association with dyslipidaemia has been also reported for *Escherichia-Shigella*, which produces LPS inflammatory mediators [9,89]. *Eggerthella* was observed to be more abundant among individuals with increased blood TG levels [6,8,13]. At least some species from this genus have been reported as modifiers of the intestinal bile acid pool [90]. The genus *Bacteroides* is diverse and comprises bacteria producing SCFAs that could potentially reduce serum cholesterol [31]. The association patterns between *Bacteroides* and metabolic traits like obesity and hyperlipidaemia vary across different studies, probably due to the high diversity of the genus [8,29,31,52,56]. Human gut methanogens, including *M. smithii*, have been found to be positively correlated with circulating cholesterol levels [15,91]. The genus *Veillonella* has been found among taxa enriched in hypercholesterolaemia. The family *Veillonelaceae* comprises lactate-degrading bacteria that are related to physical performance rates in athletes. The species *V. atypica* improved performance rates by facilitating exercise-induced lactate conversion into propionate, which supports a potential role of *Veillonella* in lipid metabolism [92].

### 4.3. Confounding Effects of Gender

Blood lipid profiles vary by gender. In general, women tend to have higher levels of serum HDL and apolipoprotein A, whereas men tend to have higher levels of circulating TG, LDL, and apolipoprotein B compared to women [93]. Gender-related disparities in serum lipid levels are primarily attributed to the sex hormone-mediated regulation of lipid metabolism, especially in the liver, the distribution of adipose tissue depots, fatty acid turnover, and other physiological factors [94]. Recently, gender-related differences in gut microbiome composition have been recognized as one of the factors contributing to the divergence in lipid metabolism between men and women [95,96]. The differential regulation can be partially explained by the two-way interaction between gut bacteria and sex hormones, as well as differences in primary and secondary bile acid levels between genders [96,97]. Therefore, gender can serve as a significant confounder for correlations between host lipid profiles and gut bacteria [87,97,98,99].

Although the average microbial alpha diversity in men was similar to that in women, and there were no significant differences in the overall gut microbiome composition, we observed significant differences in the abundance of several lipid-related bacteria between men and women. These differences included species correlated with favourable lipid profiles from the *Lachnospiraceae* family, such as *L. bacterium* and *L. bacterium Choco86*, as well as *L. eligens* and *R. intestinalis*, which are associated with lower HDL and higher TG levels. All four species were less abundant among women. The gender-specific regulation of *Lachnospiraceae* abundance has been previously described in a high-fat-diet mouse model, and these effects were linked to differences in bile acid metabolism between genders [100]. Another butyrate producer, *I. butyriciproducens*, was also less abundant among men, although it is unclear whether such differences in abundance between genders have been observed in other studies. Gender-specific differences in abundance have also been reported for species from the genera *Christensenella* and *Bacteroides*, although the functional causes of these gender-related differences in abundance and their relevance to lipid metabolism remain unclear [64,95].

However, it is important to note that our study was inadequately sized to draw conclusions about gender-specific correlations among bacterial taxa. Therefore, we cannot determine whether there is any biological explanation for the observed differences in abundance between men and women for these taxa. Additionally, it should be acknowledged that the women in our cohort had higher serum cholesterol levels and lower TG levels than the men. It cannot be ruled out that the detected correlations between lipid levels and differentially abundant species could be spurious findings confounded by gender.

### 4.4. Impact of Diet-Related Factors

Diet shapes the gut microbiome and the host’s lipid metabolism, and the shared modulation can shift associations [25,26,101,102,103]. Thet consumption of fibre-rich products correlates with reduced serum LDL and TG levels. Dietary fibre influences the proportions of circulating lipid fractions by slowing gastric emptying, delaying lipid absorption, inducing satiety, and increasing bile acid excretion [25]. On the contrary, dietary refined sugars and fats lower serum HDL while increasing circulating TG levels [25,102,103]. As a potent regulator of gut microbiome composition, diet can rapidly reshape gut microbial communities. Fibre promotes the growth of bacteria, such as SCFA producers *Bifidobacterium*, *Faecalibacterium*, and *Roseburia*, while refined sugars and fats that are high in the Western-type diet are preferred by *Enterobacteriaceae, Alistipes*, *Odoribacter*, and *Bacteroides* [27,55,80,104,105,106]. Changes induced by dietary fats depend on the quality of the fats [54,57,107]. Polyunsaturated fatty acids (PUFAs) can be considered as healthy fats with lipid-lowering and anti-inflammatory properties. A higher intake of PUFAs has been positively correlated with an abundance of *Lachnospiraceae*, *Bifidobacterium*, *Roseburia*, and *Lactobacillus* [57,108].

In our study, the consumption of fish products had the most notable impact on blood cholesterol levels and the relative abundances of bacterial species. A diet rich in fish products correlates with an improved blood cholesterol profile and alleviates metabolic syndrome [109]. Here, supplementation with salmon modulated serum lipid levels and increased the *Erysipelotrichaceae* family, *Eggerthella* genus, and *R. hominis* species relative abundances. Similarly, participants who included fish in their habitual diet more often were characterized by a higher *A. muciniphila* abundance and improved lipid profiles. Supplementation with fish-derived PUFAs increases the abundances of beneficial taxa like *Roseburia* species and *A. muciniphila* in the human gut. Nevertheless, the functional pathways linking bacteria, PUFAs, and the regulation of lipids are still under investigation [107,108]. Therefore, it is hard to judge whether the negative correlations with lower cholesterol for these taxa were due to the impact of diet, the functional contribution of bacteria, or combined effects.

Other components of the habitual diet also potentially regulated the abundances of several lipid-associated taxa. Species from the genus *Bacteroides* correlated with sugary foods, sweetened pastries, and cereal-based products, including refined flour. The increased abundance of *Bacteroides* among participants who choose foods with refined sugars is likely due to *Bacteroides*’ preference towards a diet rich in animal-derived proteins and fats and high in refined sugars [110,111]. However, consuming these products was unlikely a mediator of the observed correlations between the *Bacteroides* species and lipid profiles. Furthermore, it has not been proven that refined sugars significantly contribute to developing dyslipidaemia [103]. On the other hand, the *Peptostreptococcaceae* family is among the taxa enriched by a Mediterranean-type diet that is high in fruits and vegetables and low in animal fats [110]. Since this family was more abundant among participants consuming more poultry and fruits, we cannot exclude that lower cholesterol levels among participants with a higher abundance of *Peptostreptococcaceae* are associated with healthier food choices.

### 4.5. Study Limitations

Our study had several major limitations. The data for this study were derived from a limited number of participants. Such small cohorts are susceptible to bias and lack representativeness of the population, which can influence the direction and effect size of the detected correlations. Furthermore, this analysis was conducted in the context of a dietary intervention study with the potential to impact both lipid profiles and the composition of gut communities. Therefore, without further validation in larger cohorts, our findings cannot be generalized to the broader population.

Including additional portions of fish and meat products into the habitual diet had influenced at least some of the associations, obscuring the true relationships between the bacterial taxa and blood lipid profiles, and even more so because the number of included participants was relatively small. The restricted number of participants, high inter-individual variation, and relatively low strength of correlations between the taxa and blood lipid levels could have further lowered the possibility of detecting existing relationships, as well as having potentially increased the probability of random correlations due to the high rate of shared factors shaping both the gut microbiome composition and blood lipid profiles. The fact that none of the associations retained significance after correction for multiple tests emphasizes this issue [112]. Apart from that, the information on habitual diet collected with the food frequency questionnaire needed to be more extensive and include questions on the cooking approach. Food processing and cooking methods change the nutrient availability for the gut microbiome and can modulate the impact of the same food item [104]. The taxonomic and functional composition of the gut microbiome communities can change in response to dietary changes over short period of time [55]. Therefore, more detailed data on habitual diet collected on a daily basis would have improved the resolution and facilitated the interpretation of the analyses. Although the participants were asked to keep their dietary habits unchanged, variations in the consumed foods over the study period cannot be excluded. Reporting errors could have introduced additional bias, particularly for foods considered unhealthy [113]. The gut microbiome is a highly dynamic system, with intra-individual variation reaching up to 23% [114]. Furthermore, gut communities actively respond to short-term dietary changes [55]. Our study had a relatively short duration. Therefore, follow-up studies conducted over a longer period, exceeding 11 weeks, could be valuable to recognize serum lipid-related taxa from those showing random associations due to temporal variations in both lipid levels and taxa abundance. Additionally, sampling the gut microbiota over a more extended timeframe could help to assess the role of transient species, such as food-related *S. thermophilus*.

The under-representation of men in the cohort hindered a comprehensive assessment of gender-specific correlations. Only baseline BMI data for the participants were available; however, follow-up data would have been more suitable for the study’s objectives. Additionally, crucial information regarding several relevant lifestyle factors, such as physical activity, was missing. Physical exercise has been shown to improve lipid turnover and reduce serum cholesterol and triglyceride levels. Moreover, it can alter the composition of the gut microbiome and the spectrum of produced microbial metabolites [115]. Consequently, the absence of data on physical activity complicates the interpretation of the results obtained. Furthermore, there were variations in the collection times between the faecal and blood samples, with discrepancies of up to one day. These variations could potentially have led to misalignments in the lipid measurements and gut microbiome profiles. Lastly, it would have been advantageous to exclude participants with a genetic predisposition to dyslipidaemia [6,116]. This exclusion could have minimized the possibility of biased correlations between the serum lipid profiles and gut microbiome composition.

## 5. Conclusions

To conclude, although not without limitations, our study supports the role of the gut microbiome as an essential regulator of circulating lipid levels. Our findings confirm correlations with serum lipid profiles that were previously observed for several well-known microbial regulators of host metabolism; they also highlight the recently described, potentially novel player *M. timonensis* that regulates the host’s cholesterol turnover. However, we also detected some contradictory associations that could potentially be explained by the study design. The study was based on a very small and highly heterogenous group of participants. Apart from that, we cannot exclude any confounding effects of the dietary intervention. The observed correlations do not necessarily reflect causal relationships between members of the gut communities and the hosts’ serum lipid profiles. Therefore, further larger-scale studies involving participants selected based on their age, BMI, and lifestyle factors like physical activity in the background of a controlled diet (particularly with the dietary intake of probiotics) should help to stratify the most significant correlations and decipher the confounding factors. More precise quantification of the target taxa along with the classification of bacterial lineages representing the same species and metabolic profiles of participants could help to clarify the contradictory findings observed for several taxa. Accumulating data on association patterns could further guide in-depth functional studies to pinpoint exact interaction mechanisms and factors shifting the balance between different pathways leading to different network results on human metabolism.

## Figures and Tables

**Table 1:** General characteristics of the participants involved in the study.

Characteristic	Mean ± SD	Mean ± SD (Men/Women)	*p*-Value
Men/women	7/16	-	-
Age (years)	40.3 ± 10.8	36.3 ± 6.3/42.0 ± 12.0	>0.05
BMI (kg/m^2^)	26.3 ± 5.01	26.7 ± 4.5/26.1 ± 55.3	>0.05
Blood lipid profile			
Total Ch (mmol/L)	5.15 ± 0.90	4.5 ± 0.85/5.43 ± 0.79	0.018
HDL (mmol/L)	1.52 ± 0.43	1.3 ± 0.19/1.61 ± 0.47	>0.05
LDL (mmol/L)	2.95 ± 0.78	2.6 ± 0.69/3.11 ± 0.79	>0.05
Non-HDL Ch (mmol/L)	3.63 ± 0.93	3.20 ± 0.90/3.82 ± 0.90	>0.05
TG (mmol/L)	1.23 ± 0.59	1.30 ± 0.90/1.20 ± 0.42	>0.05

Self-reported data on age, body height, and weight were derived from questionnaires completed during recruitment. Blood lipid measurements were derived from samples donated during each visit. Differences between men and women were assessed with the Wilcoxon rank sum test, and *p*-values < 0.05 were assumed to be significant. Total Ch—total cholesterol, HDL—high-density lipoprotein, LDL—low-density lipoprotein, non-HDL Ch—calculated as HDL subtracted from total Ch, TG—triglyceride, SD—standard deviation.

## Data Availability

Sequencing data files containing raw reads have been deposited at the European Nucleotide Archive (ENA) database under study accession number PRJEB43699.

## Supplementary Materials

The following supporting information can be downloaded at https://www.mdpi.com/article/10.3390/microorganisms11112656/s1: Figure S1: Variation in circulating lipid levels among study participants; Figure S2: Variation in alpha or inner diversity of gut microbiome across eight visits; Figure S3: Association between alpha diversity of gut microbiome with participant-specific factors; Figure S4: The difference in microbiome diversity between participants with “high” and “low” levels of circulating lipids; Figure S5: Correlation between indices of microbiome richness and circulating lipid levels; Figure S6: Percentage of variance explained by the first ten principal components in PCA based on the gut microbiome composition of participants; Figure S7: The PCA plot showing clustering patterns of samples based on microbiome composition; Figure S8: Amount of variation explained by principal components and contribution of lipid variables to each principal component; Figure S9: Variance partitioning of microbiome composition at the genus and family levels; Figure S10: PCA plot showing sample separation by circulating HDL levels according to participants’ gut microbiome profiles; Figure S11: Impact of consumption of intervention food products on serum lipid levels; Figure S12: Heatmap showing a correlation between general patterns of the habitual diet of study participants and the gut microbiome composition; Figure S13: PCA-based clustering of samples of the gut microbiome collected before and after each intervention period; Figure S14: Changes in the relative abundance of taxa associated with consumption of three study products; Table S1: Correlation between measurements of circulating lipids and indices characterizing richness of gut microbiome; Table S2: General description of participants ascribed to group with “high” or “low” levels of circulating lipids; Table S3: Top ten taxa contributing to the first two principal components in the PCA based on microbiome profiles of participants; Table S4: Coefficients indicating correlation between microbial taxa and lipid measurements; Table S5: Correlations between abundance of taxa and major cofactors, BMI, age, and gender.
