# Supplementary material for: Variations in the Relative Abundance of Gut Bacteria Correlate with Lipid Profiles in Healthy Adults
Source: Microorganisms. 2023 Oct 28;11(11):2656. doi: 10.3390/microorganisms11112656 (PMC10673050; doi:10.3390/microorganisms11112656)
Supplement: Supplementary file 1 [file microorganisms-11-02656-s001.zip › Figure S1.pdf]

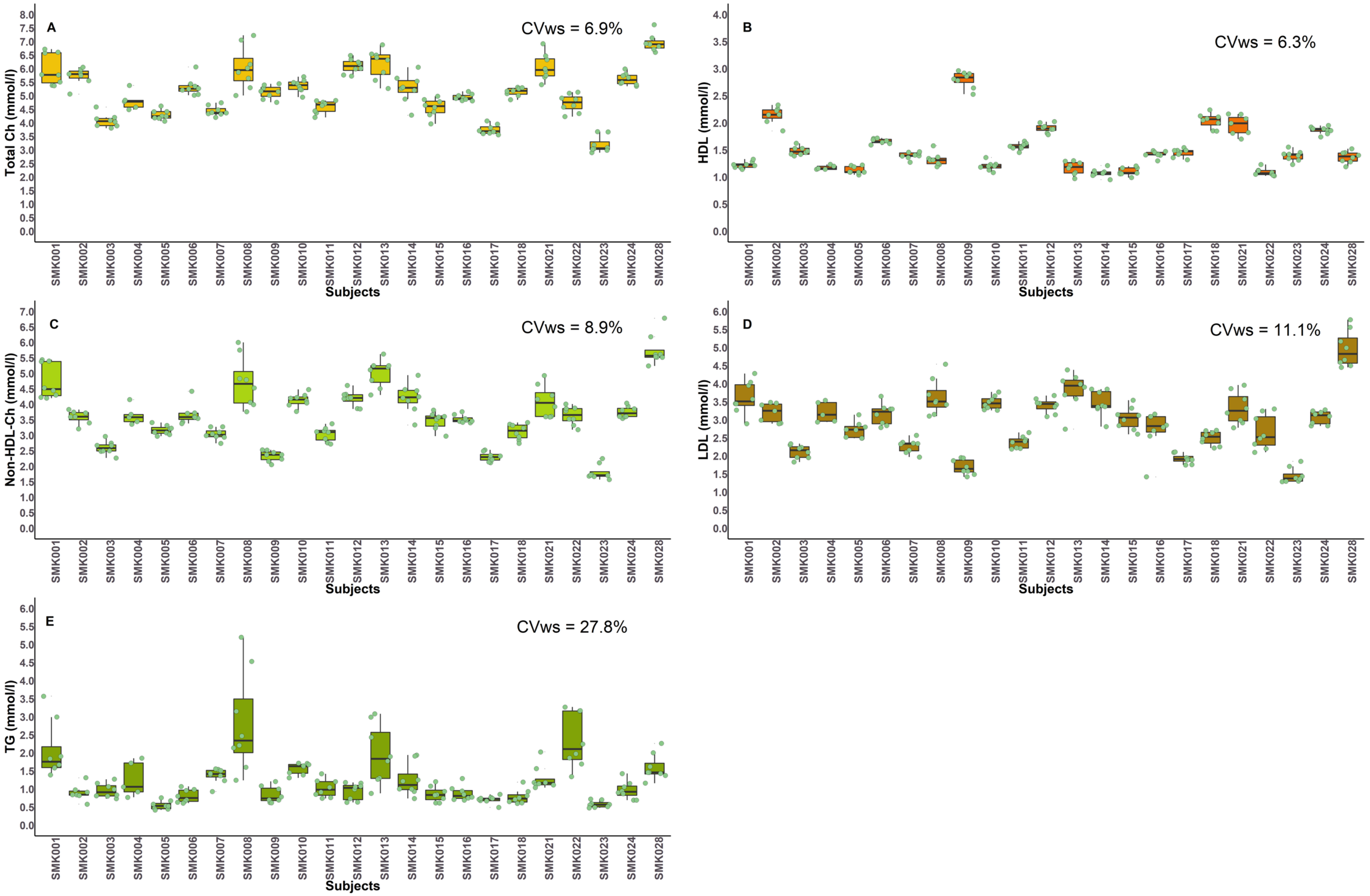

Figure S1. Variation in circulating lipid levels among study participants. Plots for each lipid parameter have been arranged as follows: **(A)** measurements of total cholesterol, **(B)** measurements of HDL, **(C)** measurements of Non-HDL Ch, **(D)** measurements of LDL, **(E)** measurements of TG in mmol/l. Boxes in the diagrams indicate the interquartile range (IQR) between the first (25 %) and third (75 %) quartiles. The line dividing each box indicates the median. Upper whiskers indicate dots within 1.5x the interquartile range above the third quartile, and lower whiskers are 1.5x the interquartile range below the first quartile. Total Ch—total cholesterol; HDL—high-density lipoproteins; Non-HDL Ch—non-high density lipoproteins, calculated as HDL subtracted from TCh; LDL—low-density lipoproteins; TG—triglycerides; CV_{ws}—within-subject coefficient of variation calculated using root mean square approach.
