# Supplementary material for: Variations in the Relative Abundance of Gut Bacteria Correlate with Lipid Profiles in Healthy Adults
Source: Microorganisms. 2023 Oct 28;11(11):2656. doi: 10.3390/microorganisms11112656 (PMC10673050; doi:10.3390/microorganisms11112656)
Supplement: Supplementary file 1 [file microorganisms-11-02656-s001.zip › Figure S10.pdf]

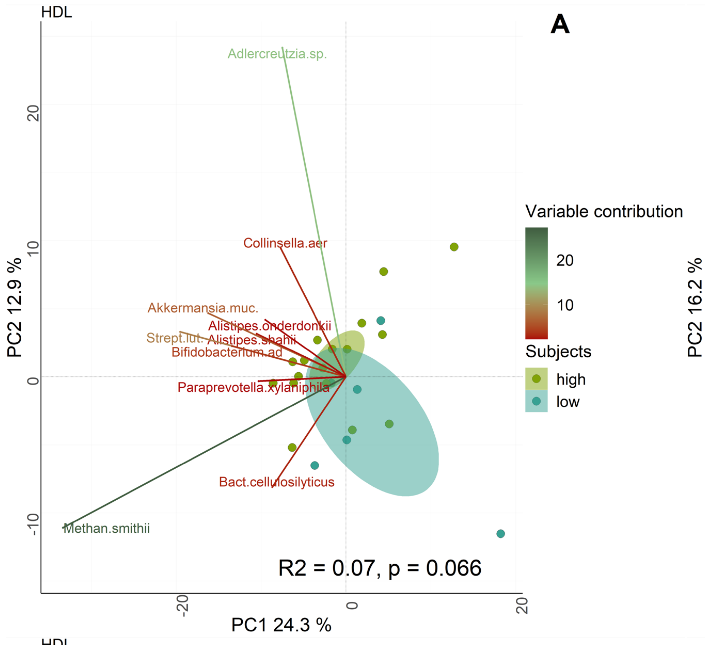

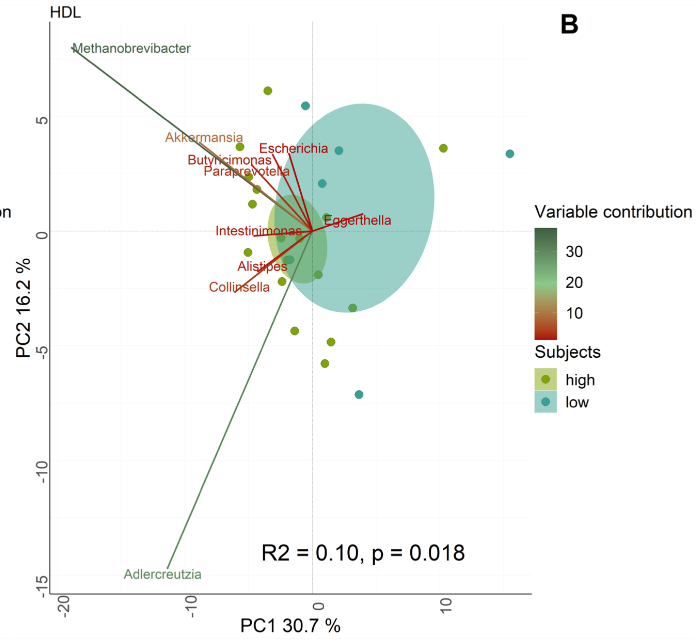

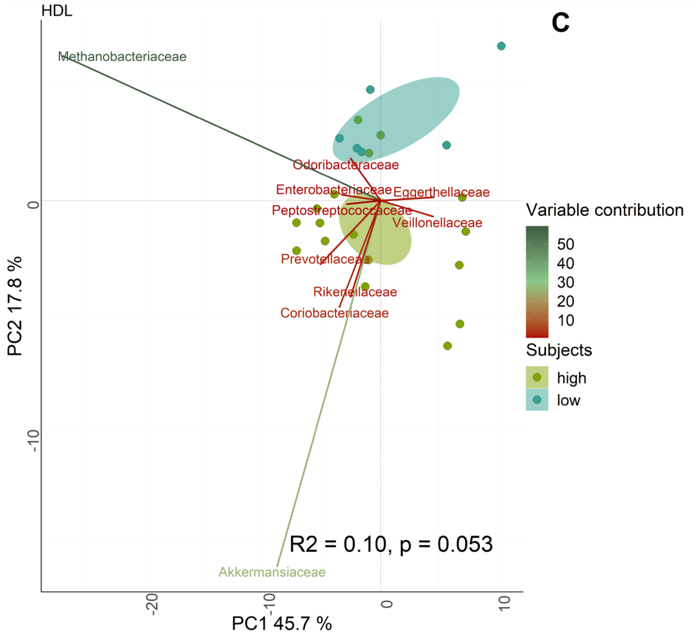

D

adonis	R^2	p-value
	Species	
Total_Ch	0.051	0.286
HDL	0.07	0.066
LDL	0.03	0.856
Non-HDL-Ch	0.025	0.95
TG	0.038	0.616
	**Genus**	
Total_Ch	0.086	0.04
HDL	0.1	0.018
LDL	0.038	0.597
Non-HDL-Ch	0.036	0.659
TG	0.044	0.439
	**Family**	
Total_Ch	0.069	0.187
HDL	0.1	0.053
LDL	0.027	0.0719
Non-HDL-Ch	0.023	0.803
TG	0.021	0.866

Figure S10. PCA plot showing sample separation by circulating HDL levels according to participants' gut microbiome profiles. The clustering of participants is shown at three taxonomic levels: **(A)** at the species, **(B)** at the genus and **(C)** at the family level. Arrows in the PCA plot indicate contribution to principal components of the top ten taxa at each taxonomic level. The colours of the dots indicate subjects with "high" or "low" average circulating levels of HDL. The two categories of lipid levels, "high"and "low", were defined according to the following cut-off value: HDL ≥ 1.2 (mmol/l). Ellipses correspond to 95 % confidence intervals. Only the first two principal components (PC) explaining the highest proportion of variation are shown. **(D)** The table includes coefficients and p-values describing differences between the two groups regarding the gut microbiome composition. Differences between groups were assessed with the *adonis* function in the R package "vegan" (999 permutations). R^2—coefficient expressing variation in distances explained by the grouping being tested, p—p-value < 0.05 were assumed as significant, HDL - high-density lipoproteins, TCh—total cholesterol, Non-HDL-Ch—non-HDL cholesterol, LDL—low-density lipoproteins and TG—triglycerides.
