# Supplementary material for: Variations in the Relative Abundance of Gut Bacteria Correlate with Lipid Profiles in Healthy Adults
Source: Microorganisms. 2023 Oct 28;11(11):2656. doi: 10.3390/microorganisms11112656 (PMC10673050; doi:10.3390/microorganisms11112656)
Supplement: Supplementary file 1 [file microorganisms-11-02656-s001.zip › Figure S11.pdf]

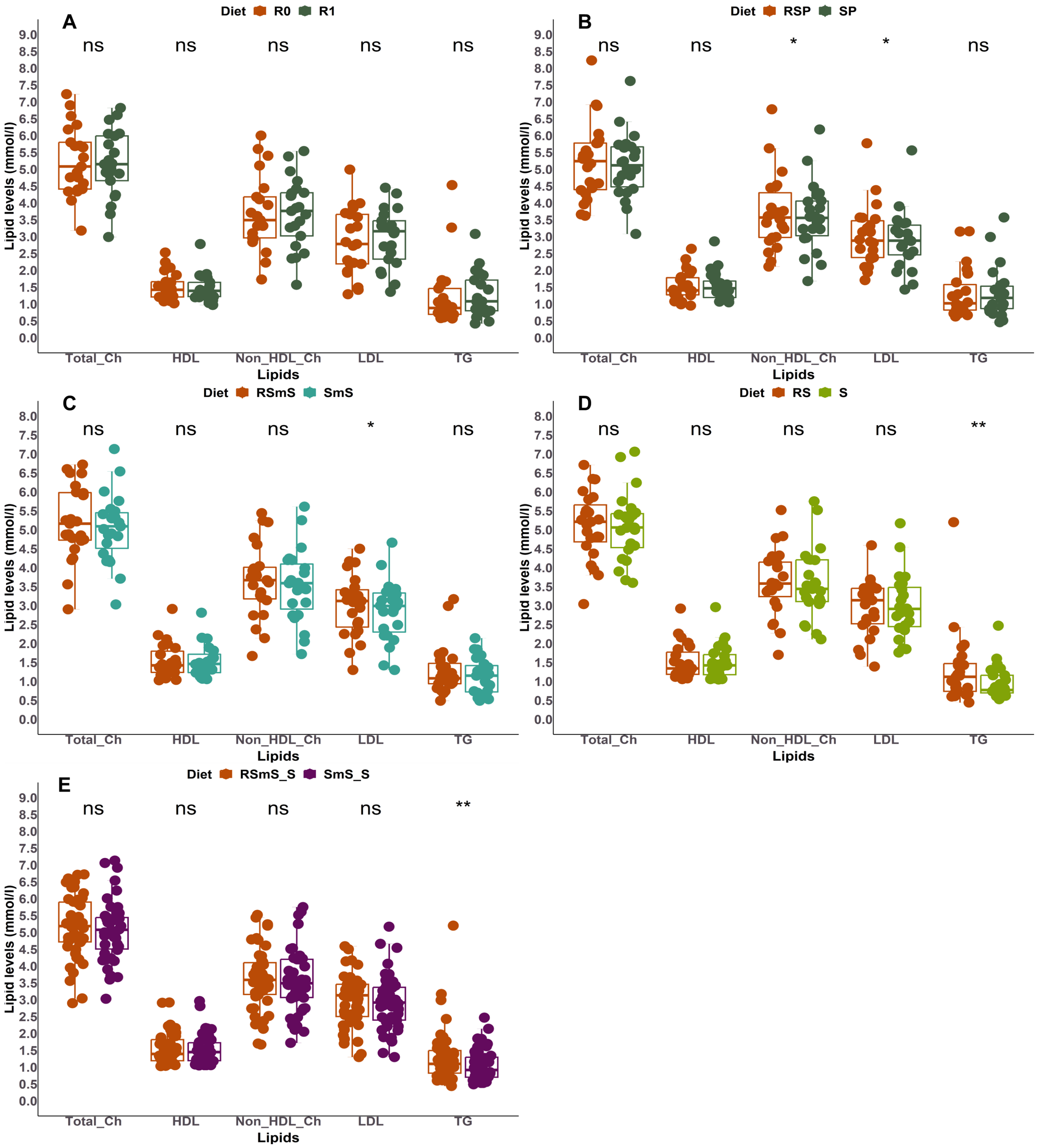

Figure S11. Impact of consumption of intervention food products on serum lipid levels. **(A)** Differences between lipid measurements at the first visit (R0) compared to the last study visit two weeks after the last intervention period (R1). Differences in serum lipid levels between samples collected before and after each intervention week are plotted by specific product as follows: **(B)** smoked pork (SP), **(C)** smoked salmon (SmS), **(D)** salmon (S, provided fresh, cooked at will), **(E)** salmon and smoked salmon merged as a single product (SmS_S). The significance of differences was assessed with the Wilcoxon signed ranks test, assuming $p < 0.05$ as the significance threshold. R—reference samples; ns—non-significant; *—$p < 0.05$; **—$p < 0.01$. Total Ch—total cholesterol, Non-HDL-Ch—non-HDL cholesterol, LDL—low-density lipoproteins and TG - triglycerides.
