# Supplementary material for: Variations in the Relative Abundance of Gut Bacteria Correlate with Lipid Profiles in Healthy Adults
Source: Microorganisms. 2023 Oct 28;11(11):2656. doi: 10.3390/microorganisms11112656 (PMC10673050; doi:10.3390/microorganisms11112656)
Supplement: Supplementary file 1 [file microorganisms-11-02656-s001.zip › Figure S13.pdf]

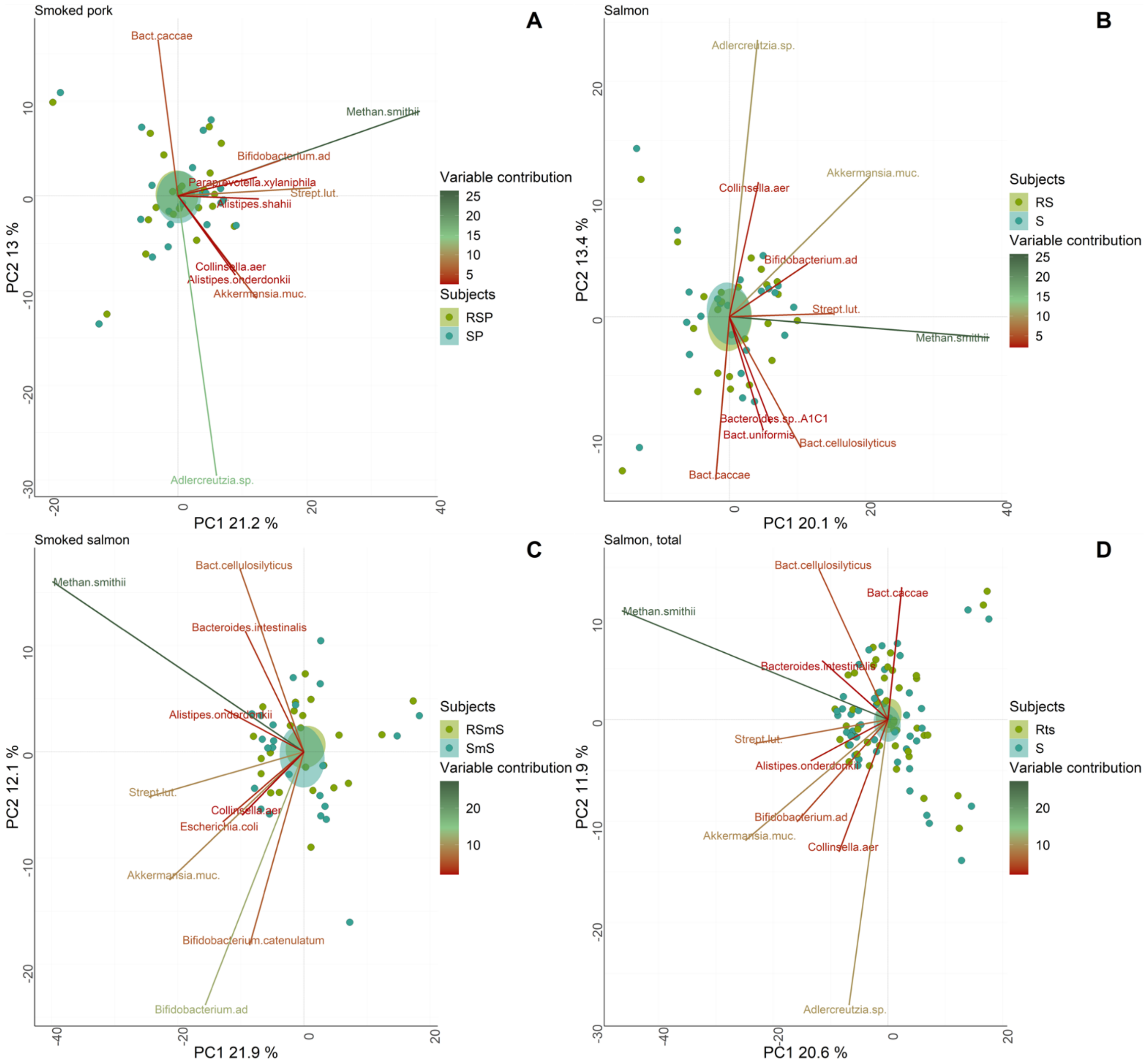

Figure S13. PCA-based clustering of samples of the gut microbiome collected before and after each intervention period. Clustering of samples collected before and after each intervention week by the composition of the gut microbiome is plotted by specific product as follows: **(A)** smoked pork (SP), **(B)** salmon (S, provided fresh, cooked at will), **(C)** smoked salmon (SmS), **(D)** salmon and smoked salmon merged as a single product (SmS_S). Arrows indicate the top ten taxa contributing to the first two principal components. The colours of the arrows correspond to the contribution of each variable. Ellipses correspond to 95 % confidence intervals. Only the first two principal components (PC) explaining the highest proportion of variation are drawn in the plot.

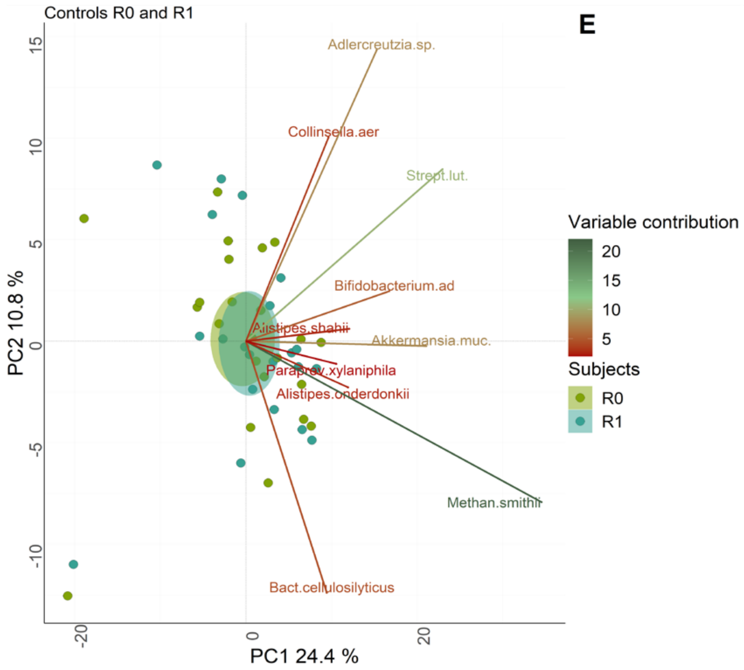

E

F

adonis	R^2	p-value
**Species**		
Controls (R0 – R1)	0.0076	0.231
Smoked pork (RSP-SP)	0.00401	0.48
Salmon (RS-S)	0.0033	0.629
Smoked salmon (RSmS - SmS)	0.0057	0.231
Salmon- total (RSmS- SmS and RS-S)	0.0027	0.239
**Genus**		
Controls (R0 – R1)	0.011	0.124
Smoked pork (RSP-SP)	0.0056	0.444
Salmon (RS-S)	0.0048	0.327
Smoked salmon (RSmS - SmS)	0.0058	0.318
Salmon- total (RSmS- SmS and RS-S)	0.0030	0.32
**Family**		
Controls (R0 – R1)	0.0067	0.398
Smoked pork (RSP-SP)	0.0054	0.307
Salmon (RS-S)	0.0033	0.738
Smoked salmon (RSmS - SmS)	0.0089	0.066
Salmon- total (RSmS- SmS and RS-S)	0.0039	0.108

Figure S13 continued. **(E)** The figure shows differences in the gut composition of the gut microbiome between samples collected before the study (R0) compared to those donated after the eighth visit of the study two weeks after the last intervention period (R1). Arrows indicate the top ten taxa contributing to the first two principal components. The colours of the arrows correspond to the contribution of each variable. Ellipses correspond to 95 % confidence intervals. **(F)** The table summarizes coefficients and p-values describing differences between the two groups regarding gut microbiome composition. Differences between groups were assessed with the *adonis* function in the R package "vegan" (999 permutations). R^2—coefficient expressing variation in distances explained by the grouping being tested, p—p-value < 0.05 were assumed as significant.
