# Supplementary material for: Variations in the Relative Abundance of Gut Bacteria Correlate with Lipid Profiles in Healthy Adults
Source: Microorganisms. 2023 Oct 28;11(11):2656. doi: 10.3390/microorganisms11112656 (PMC10673050; doi:10.3390/microorganisms11112656)
Supplement: Supplementary file 1 [file microorganisms-11-02656-s001.zip › Figure S14.pdf]

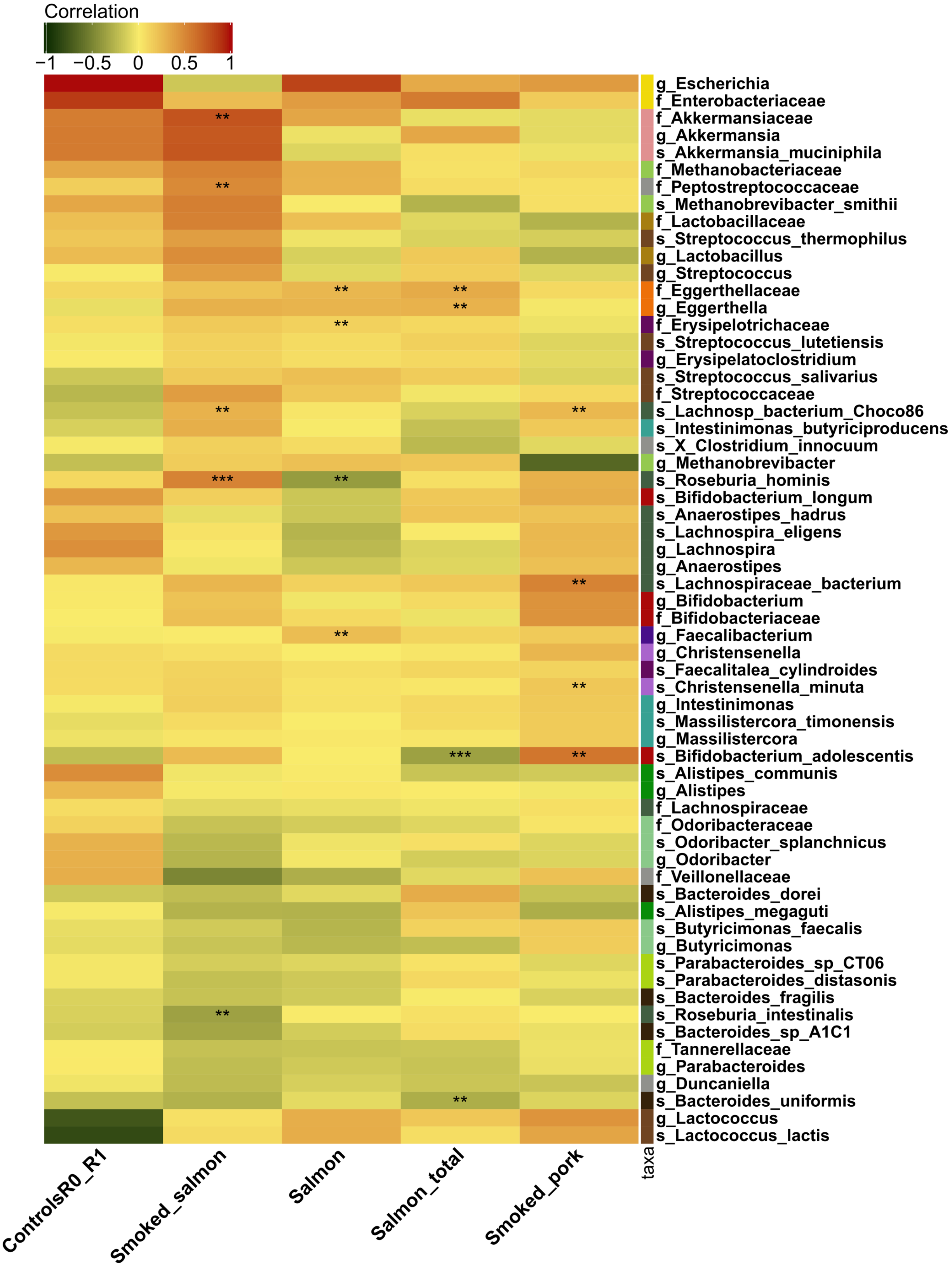

Figure S14. Changes in the relative abundance of taxa associated with consumption of three study products. The plot includes only taxa previously associated with lipid levels. The colours in the heat map reflect coefficients for each taxon calculated with Maaslin2. p-values < 0.05 were considered significant. The colours of the vertical annotation bar show relatedness among taxa. **—p-values < 0.05 and ***—p-values < 0.01; s—species; g—genus; f—family.
