# Supplementary material for: Variations in the Relative Abundance of Gut Bacteria Correlate with Lipid Profiles in Healthy Adults
Source: Microorganisms. 2023 Oct 28;11(11):2656. doi: 10.3390/microorganisms11112656 (PMC10673050; doi:10.3390/microorganisms11112656)
Supplement: Supplementary file 1 [file microorganisms-11-02656-s001.zip › Figure S2.pdf]

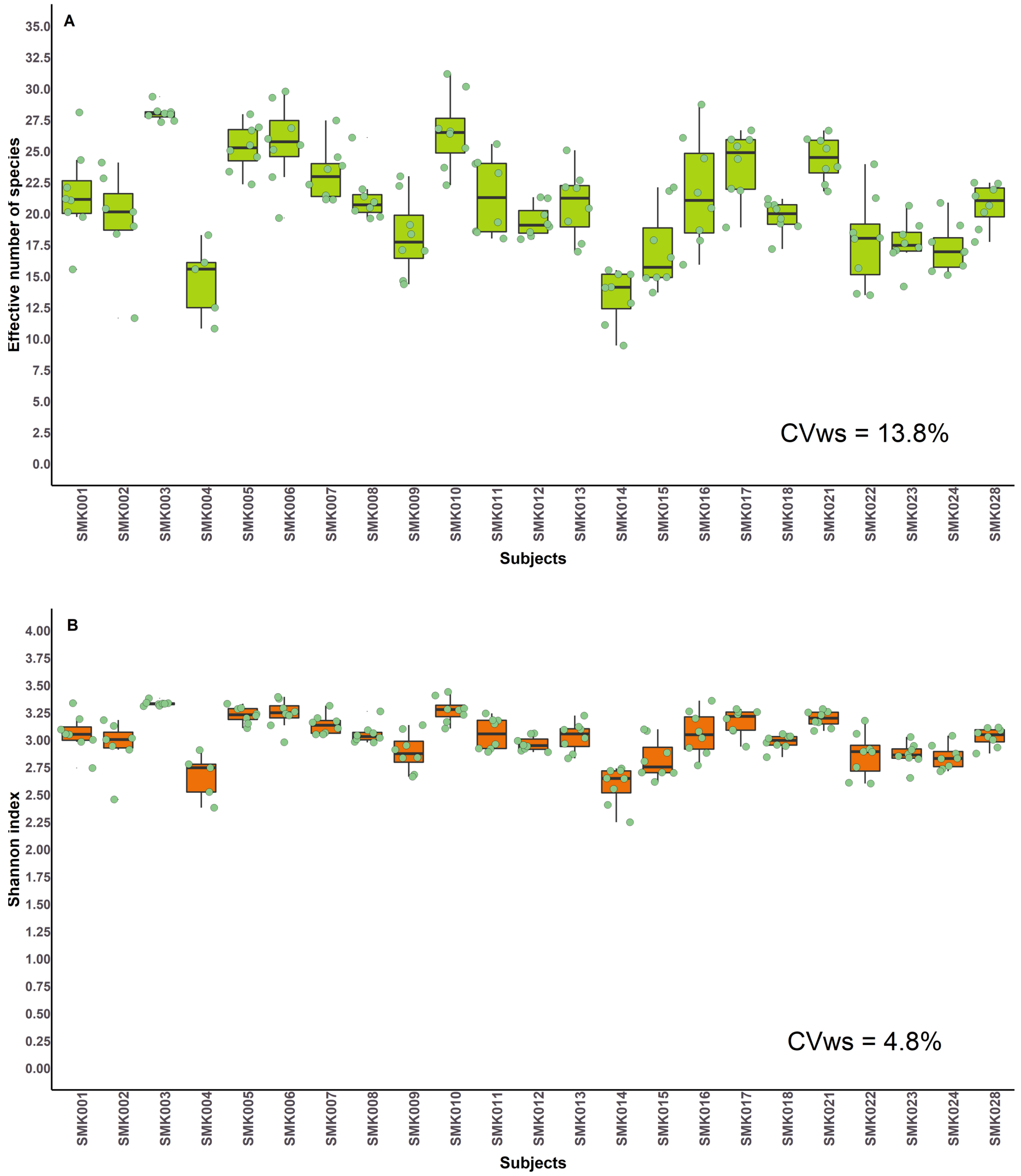

Figure S2. Variation in alpha or inner diversity of gut microbiome across eight visits. **(A)** Variation of microbiome diversity expressed as the Effective number of species. **(B)** Variation of individual-specific Shannon diversity index calculated for samples collected from each subject. Boxes in the diagrams indicate the interquartile range (IQR) between the first (25 %) and third (75 %) quartiles. The line dividing each box indicates the median. Upper whiskers indicate dots within 1.5 times the interquartile range above the third quartile, and lower whiskers are 1.5 times the interquartile range below the first quartile. The Effective number of species and Shannon index were calculated at the species level, including microbial taxa, with a median number of reads over 1000. CV_{ws}—within-subject coefficient of variation calculated using root mean square approach.
