# Supplementary material for: Variations in the Relative Abundance of Gut Bacteria Correlate with Lipid Profiles in Healthy Adults
Source: Microorganisms. 2023 Oct 28;11(11):2656. doi: 10.3390/microorganisms11112656 (PMC10673050; doi:10.3390/microorganisms11112656)
Supplement: Supplementary file 1 [file microorganisms-11-02656-s001.zip › Figure S3.pdf]

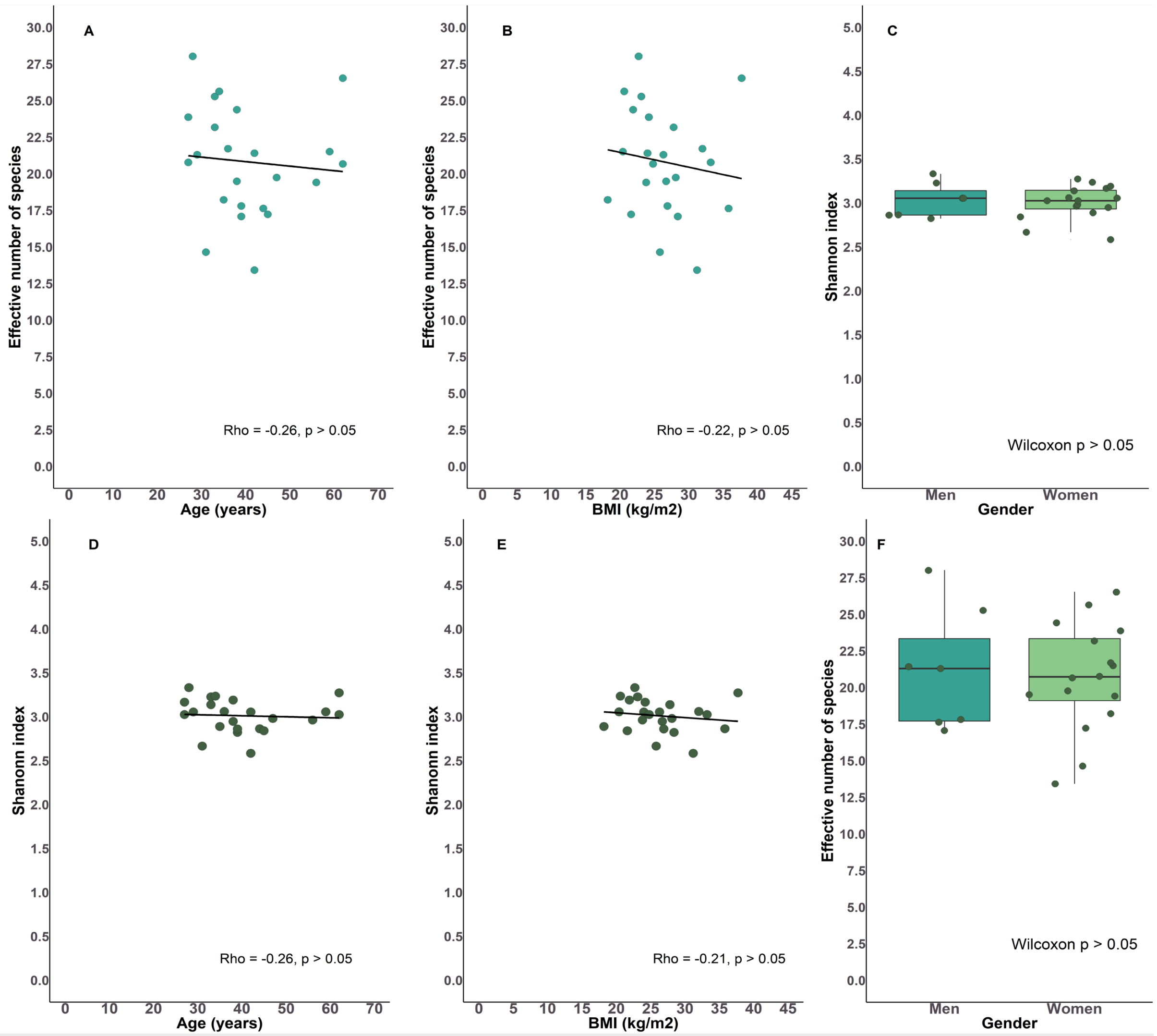


| Paramters | Effective number of species | | Shanonn's index | |
|---|---|---|---|---|
| | Coefficient | p-value | Coefficient | p-value |
| Gender (men/women) | 21.2 ± 4.17/20.7 ± 3.67* | > 0.05 | 3.03 ± 0.20/3.0 ± 0.19* | > 0.05 |
| Age (years) | -0.26 | > 0.05 | -0.26 | > 0.05 |
| BMI (kg/m$^2$) | -0.22 | > 0.05 | -0.21 | > 0.05 |

**Figure S3**. Association between alpha diversity of gut microbiome with participant-specific factors. Figures **(A)** and **(D)** show the correlation between participants' age and diversity estimates, the effective number of species and Shannon's diversity index. Figures **(B)** and **(E)** show a relationship between BMI and alpha diversity. Figures **(C)** and **(F)** show that the effective number of species and Shannon's diversity index did not differ between men and women. The table below figures contains a summary of the association results. *—average effective number of species and Shanonn's diversity index with respective standard deviations. The correlation between age, BMI and diversity estimates was assessed with Spearman's correlation. Average diversity estimates between genders were compared using the Wilcoxon rank sum test. The significance threshold was set at $p < 0.05$. Rho—Spearman's correlation coefficient; BMI—body mass index.
