# Supplementary material for: Variations in the Relative Abundance of Gut Bacteria Correlate with Lipid Profiles in Healthy Adults
Source: Microorganisms. 2023 Oct 28;11(11):2656. doi: 10.3390/microorganisms11112656 (PMC10673050; doi:10.3390/microorganisms11112656)
Supplement: Supplementary file 1 [file microorganisms-11-02656-s001.zip › Figure S4.pdf]

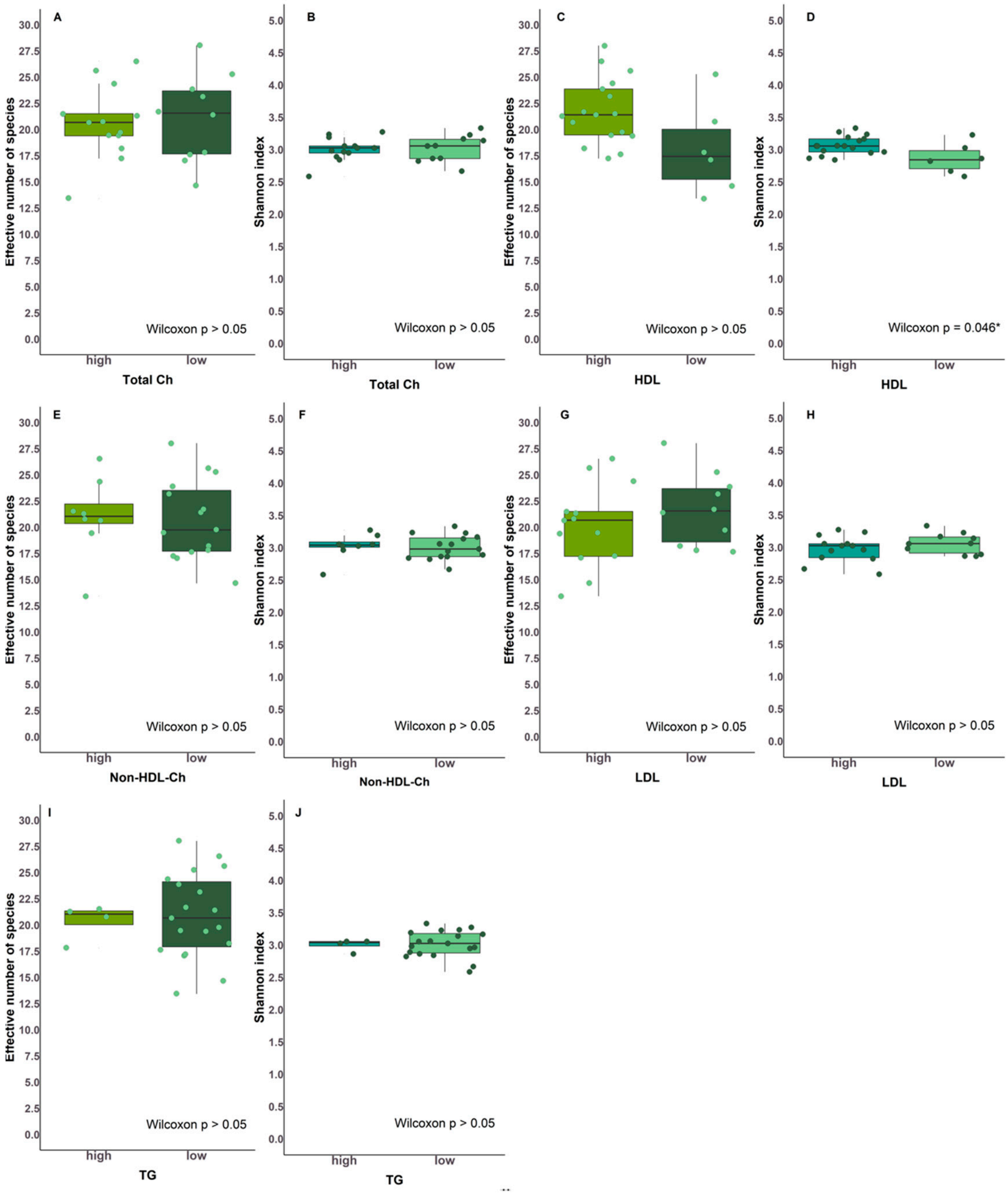

Figure S4. The difference in microbiome diversity between participants with "high" and "low" levels of circulating lipids. Alpha diversity was expressed as the effective number of species and Shannon's diversity index. **(A)** and **(B)** show difference in Shanonon's diversity index and the effective number of species between participants with "high" and "low" total Ch levels, **(C)** and **(D)** show differences in alpha diversity measures between participants with "high" and "low" HDL levels, **(E)** and **(F)** show differences in alpha diversity measures between participants with "high" and "low" Non-HDL Ch levels, **(G)** and **(H)** show differences in alpha diversity measures between participants with "high" and "low" LDL levels and **(I)** and **(J)** display differences in Shannon's diversity index and the effective number of species between participants with "high" and "low" TG levels. Wilcoxon rank-sum test was used to compare alpha diversity between participants with "high" and "low" lipid levels, assuming $p > 0.05$ as significant. Participants were assigned to the "high" or "low" lipid group using the following standard lipid reference values, which were as follows: Total Ch < 5.0 (mmol/l), HDL ≥ 1.2 (mmol/l), Non-HDL Ch ≤ 3.9 (mmol/l), LDL < 3.0 (mmol/l), TG < 1.7(mmol/l). *—unadjusted p-value, after correction for multiple testing, significance was lost ($p_{adjust} > 0.05$); Total Ch—total cholesterol; HDL—high-density lipoproteins; Non-HDL Ch—non-high density lipoproteins, calculated as HDL subtracted from TCh; LDL—low-density lipoproteins; TG—triglycerides.
