# Supplementary material for: Variations in the Relative Abundance of Gut Bacteria Correlate with Lipid Profiles in Healthy Adults
Source: Microorganisms. 2023 Oct 28;11(11):2656. doi: 10.3390/microorganisms11112656 (PMC10673050; doi:10.3390/microorganisms11112656)
Supplement: Supplementary file 1 [file microorganisms-11-02656-s001.zip › Figure S5.pdf]

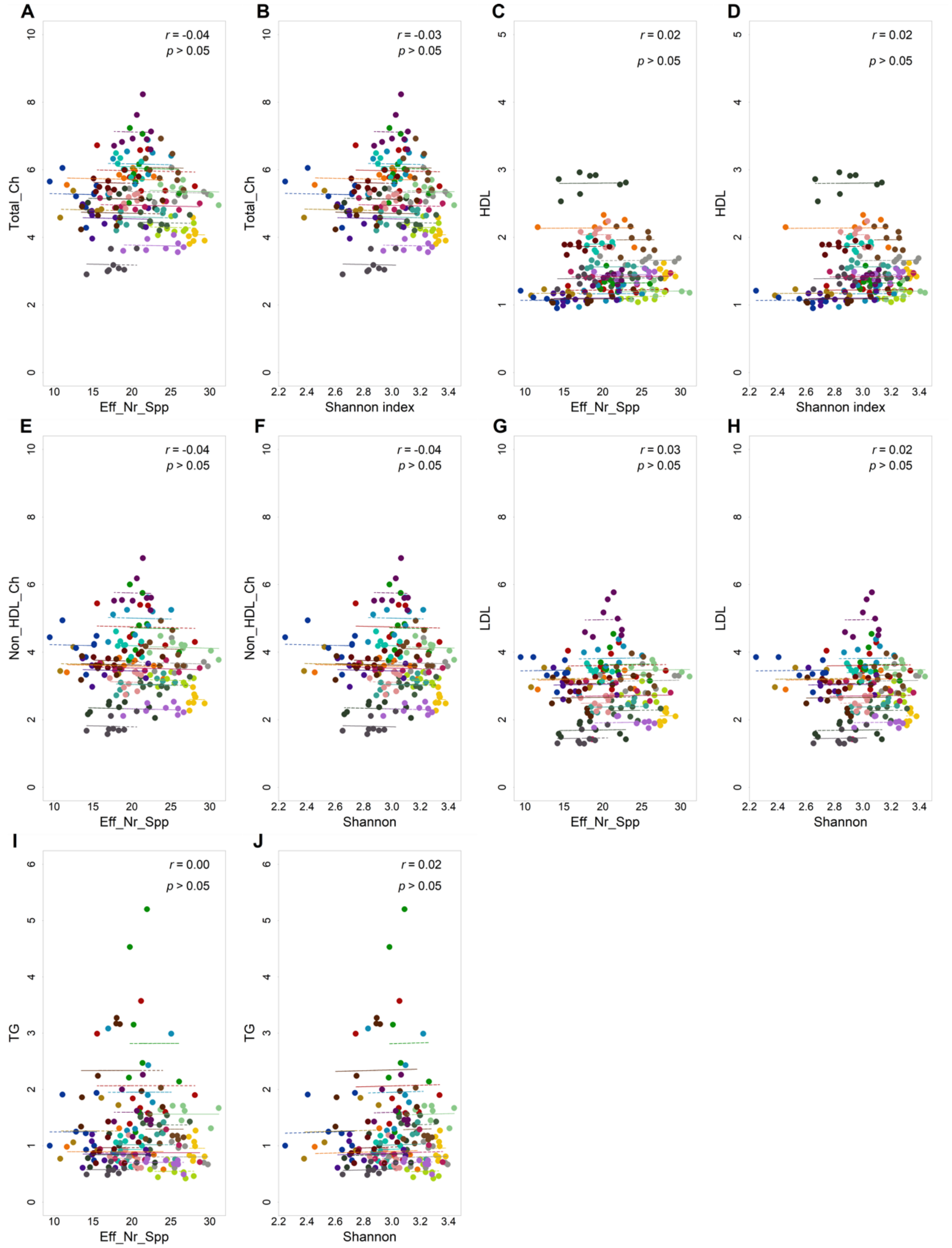

Figure S5. Correlation between indices of microbiome richness, the effective number of species, Shannon's diversity index, and circulating lipid levels. **(A)** and **(B)** show correlation between total Ch blood levels measured at eight time points with Shannon's diversity index and the effective number of species, **(C)** and **(D)** show correlation between the follow-up data on HDL levels and alpha diversity measures, **(E)** and **(F)** show correlation between the follow-up data on Non-HDL Ch levels and alpha diversity measures, **(G)** and **(H)** show correlation between the follow-up data on LDL levels and alpha diversity measures, whereas **(I)** and **(J)** reflect relationships between the follow-up measurements of TG levels and Shannon's diversity index and the effective number of species. Eff-Nr-Spp—Effective number of species; Shannon - Shannon's index; Total-Ch - total cholesterol; HDL—high-density lipoproteins, Non-HDL-Ch—non-HDL cholesterol; LDL—low-density lipoproteins, TG—triglycerides; r—rmcorr correlation coefficient representing the strength of the association, p—p-value. p-value < 0.05 was assumed to be significant. Colours correspond to samples collected from each individual.
