# Supplementary material for: Variations in the Relative Abundance of Gut Bacteria Correlate with Lipid Profiles in Healthy Adults
Source: Microorganisms. 2023 Oct 28;11(11):2656. doi: 10.3390/microorganisms11112656 (PMC10673050; doi:10.3390/microorganisms11112656)
Supplement: Supplementary file 1 [file microorganisms-11-02656-s001.zip › Figure S6.pdf]

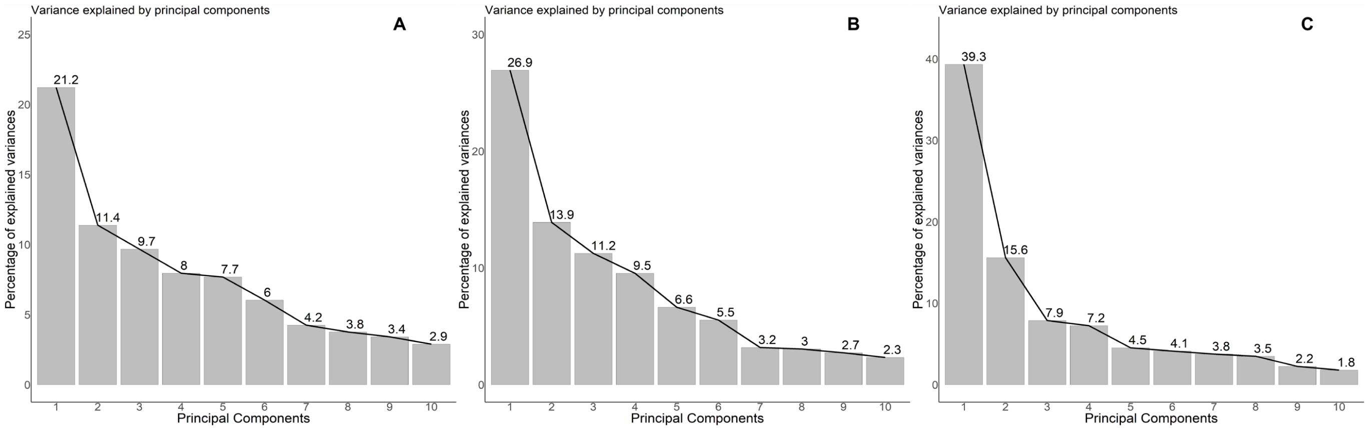

Figure S6. Percentage of variance explained by the first ten principal components in beta PCA based on the gut microbiome composition of participants. PCA was carried out at the species **(A)**, at the genus **(B)** and the family **(C)** levels. The bar plots include only the top ten principal components explaining the highest variance proportion. The numbers above bars indicate the percentage of variance explained by the respective principal component.
