# Supplementary material for: Variations in the Relative Abundance of Gut Bacteria Correlate with Lipid Profiles in Healthy Adults
Source: Microorganisms. 2023 Oct 28;11(11):2656. doi: 10.3390/microorganisms11112656 (PMC10673050; doi:10.3390/microorganisms11112656)
Supplement: Supplementary file 1 [file microorganisms-11-02656-s001.zip › Figure S7.pdf]

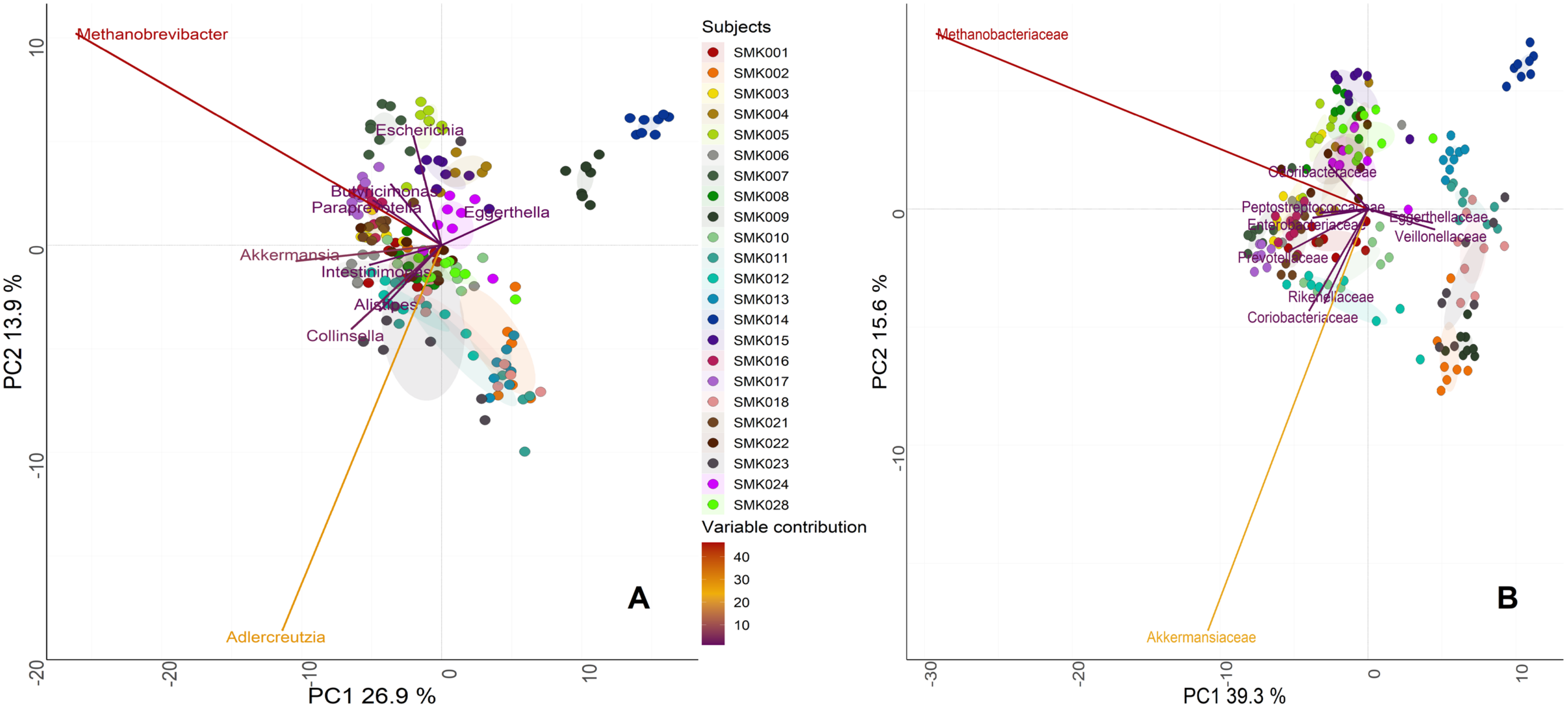


**Figure S7.** The PCA plot shows clustering patterns of samples based on microbiome composition. Sample distribution patterns **(A)** based on participant-specific gut microbiome profiles at the genus level and **(B)** based on participant-specific microbiome profiles at the family level. Arrows indicate the top ten contributing bacterial taxa. The arrows' colour corresponds to each variable's contribution to the principal axes. Ellipses show 95 % confidence intervals. Dots are coloured by the participant. The first two principal components, PC1 and PC2, explained the highest proportion of variation.
