# Supplementary material for: Variations in the Relative Abundance of Gut Bacteria Correlate with Lipid Profiles in Healthy Adults
Source: Microorganisms. 2023 Oct 28;11(11):2656. doi: 10.3390/microorganisms11112656 (PMC10673050; doi:10.3390/microorganisms11112656)
Supplement: Supplementary file 1 [file microorganisms-11-02656-s001.zip › Figure S8.pdf]

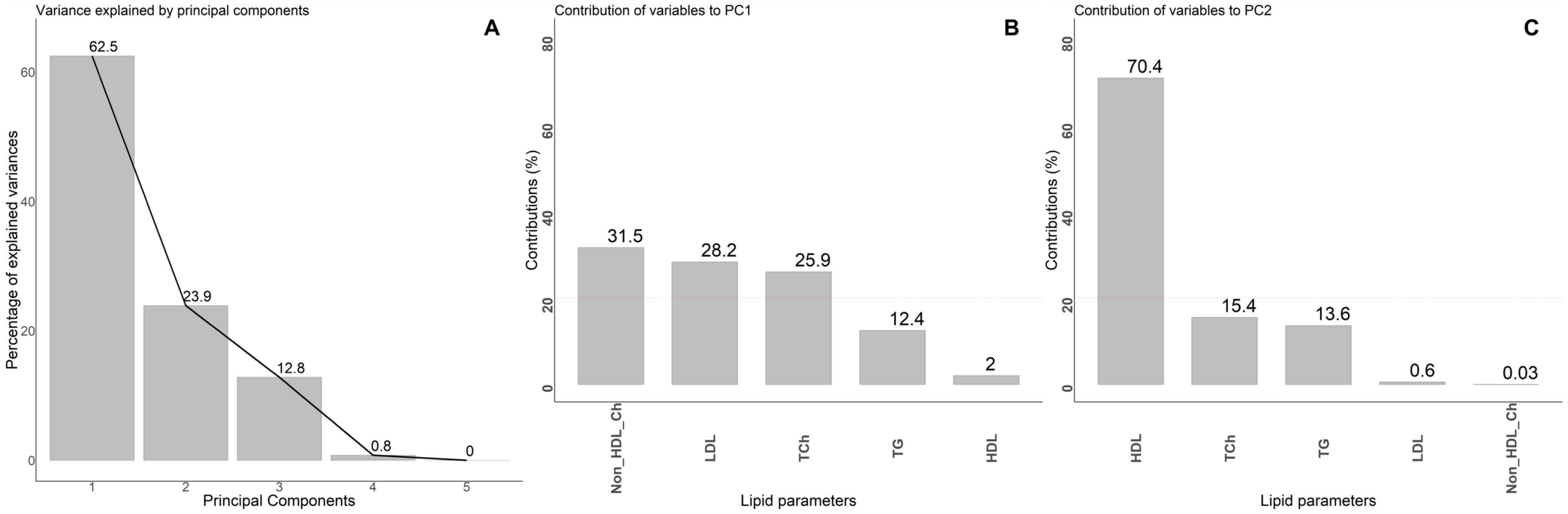

Figure S8. Amount of variation explained by principal components and contribution of lipid variables to each principal component. **(A)** The total variation among samples explained by five principal components in PCA based on lipid parameters. **(B)** shows the contribution of lipid parameters to PC1 and **(C)** to PC2. Labels above bars in the plot indicate explained variance and contribution of variables in percentages. TCh—total cholesterol; HDL—high-density lipoproteins; Non-HDL-Ch—non-high density lipoproteins, calculated as HDL subtracted from total Ch; LDL—low-density lipoproteins; TG—triglycerides.
