# Supplementary material for: Variations in the Relative Abundance of Gut Bacteria Correlate with Lipid Profiles in Healthy Adults
Source: Microorganisms. 2023 Oct 28;11(11):2656. doi: 10.3390/microorganisms11112656 (PMC10673050; doi:10.3390/microorganisms11112656)
Supplement: Supplementary file 1 [file microorganisms-11-02656-s001.zip › Figure S9.pdf]

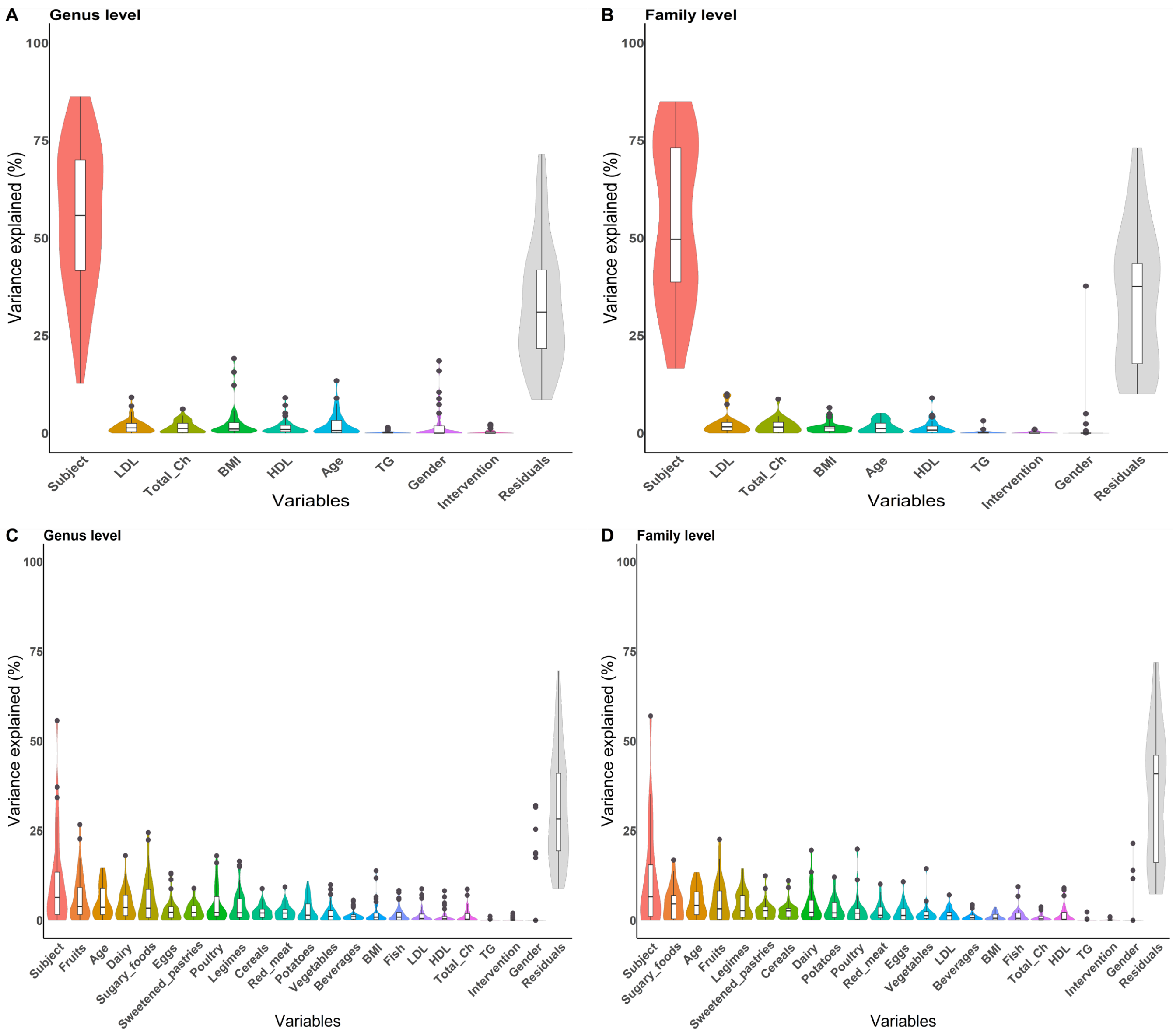

Figure S9. Variance partitioning of microbiome composition at the genus and family levels. **(A)** Variance in the relative abundance of genera explained by major participant-related confounding factors (collinearity score 0.84). **(B)** Variance explained by factors other than those related to habitual diet at the family level (collinearity score 0.90). **(C)** Variance in the relative abundance of genera explained by major product groups reflecting dietary habits included in the model (collinearity score 0.83). **(D)** Variance in microbiome composition at the family level exlained by participant-related factors, including habitual diet (collinearity score 0.91). Non-HDL Ch measurements were excluded from the analysis due to association with other lipid measurements. All models are based on each participant's complete longitudinal microbial data set. The residual group comprises undefined contributors to the variance. Total Ch—total cholesterol, HDL—high-density lipoproteins, LDL—low-density lipoproteins and TG—triglycerides, BMI—body mass index.
