# Supplementary material for: Variations in the Relative Abundance of Gut Bacteria Correlate with Lipid Profiles in Healthy Adults
Source: Microorganisms. 2023 Oct 28;11(11):2656. doi: 10.3390/microorganisms11112656 (PMC10673050; doi:10.3390/microorganisms11112656)
Supplement: Supplementary file 1 [file Figure S12.pdf › Figure S12.pdf]

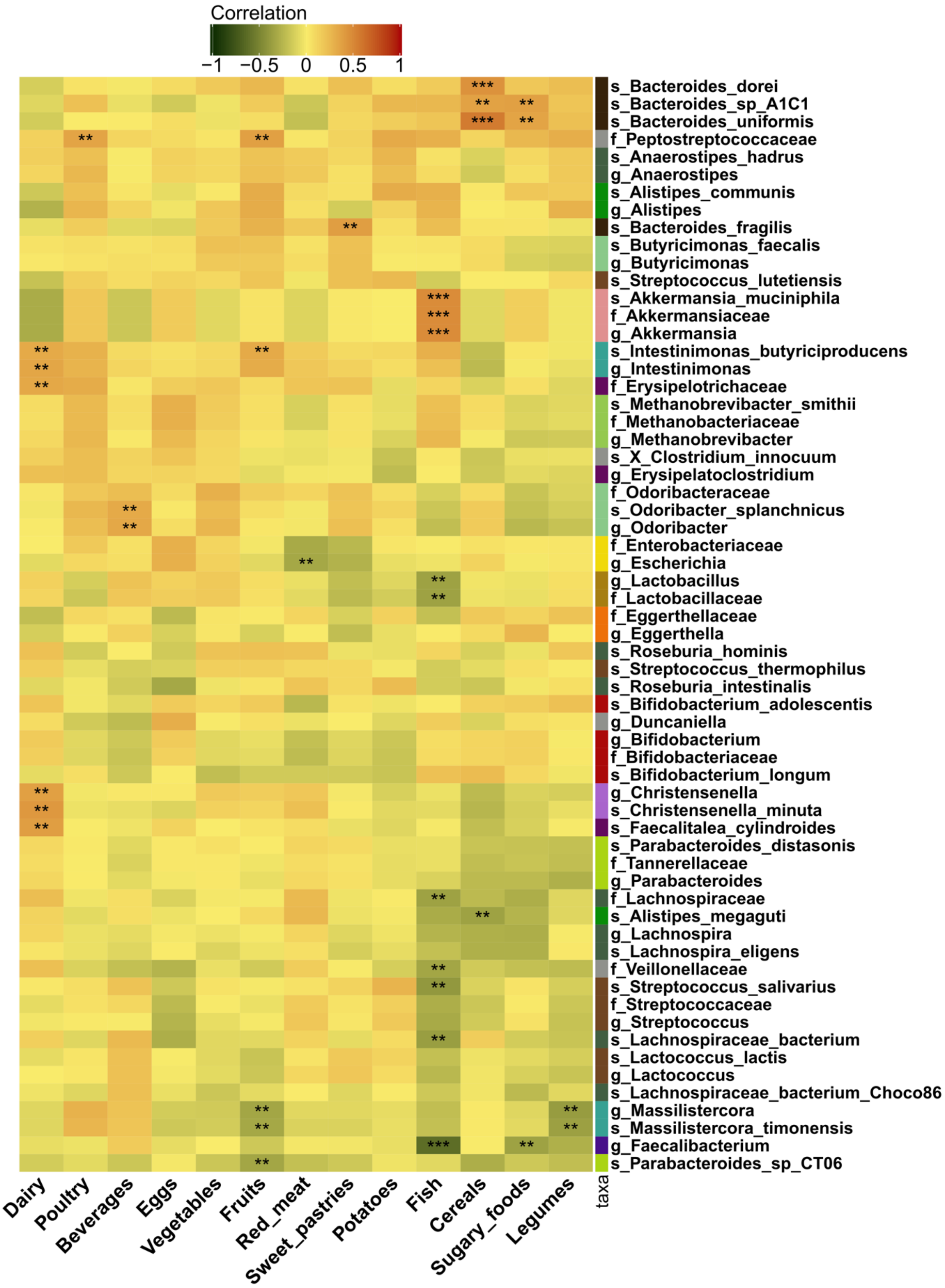

Figure S12. Heatmap showing a correlation between general patterns of the habitual diet of study participants and the gut microbiome composition. The plot includes only taxa previously associated with lipid levels. Correlations were estimated with Kendall's correlation test using ordinal data collected by food frequency questionnaires filled out at the beginning of the study. The colours of the cells correspond to Kendall's tau, and asterisks indicate p-values. The colours of the annotation bar show relatedness between taxa.** —$p < 0.05$; ***—$p < 0.01$; s—species; g—genus; f—family.
